# Copper oxide nanoparticles trigger macrophage cell death with misfolding of Cu/Zn superoxide dismutase 1 (SOD1)

**DOI:** 10.1186/s12989-022-00467-w

**Published:** 2022-05-10

**Authors:** Govind Gupta, Francesca Cappellini, Lucian Farcal, Rosalba Gornati, Giovanni Bernardini, Bengt Fadeel

**Affiliations:** 1 Division of Molecular Toxicology, Institute of Environmental Medicine, Karolinska Institutet, Nobels väg 13, Stockholm, Sweden; 2 Department of Biotechnology and Life Sciences, University of Insubria, Varese, Italy

**Keywords:** Copper oxide nanoparticles, Mitochondria, Oxidative stress, Protein misfolding

## Abstract

**Background:**

Copper oxide (CuO) nanoparticles (NPs) are known to trigger cytotoxicity in a variety of cell models, but the mechanism of cell death remains unknown. Here we addressed the mechanism of cytotoxicity in macrophages exposed to CuO NPs versus copper chloride (CuCl_2_).

**Methods:**

The mouse macrophage cell line RAW264.7 was used as an in vitro model. Particle uptake and the cellular dose of Cu were investigated by transmission electron microscopy (TEM) and inductively coupled plasma mass spectrometry (ICP-MS), respectively. The deposition of Cu in lysosomes isolated from macrophages was also determined by ICP-MS. Cell viability (metabolic activity) was assessed using the Alamar Blue assay, and oxidative stress was monitored by a variety of methods including a luminescence-based assay for cellular glutathione (GSH), and flow cytometry-based detection of mitochondrial superoxide and mitochondrial membrane potential. Protein aggregation was determined by confocal microscopy using an aggresome-specific dye and protein misfolding was determined by circular dichroism (CD) spectroscopy. Lastly, proteasome activity was investigated using a fluorometric assay.

**Results:**

We observed rapid cellular uptake of CuO NPs in macrophages with deposition in lysosomes. CuO NP-elicited cell death was characterized by mitochondrial swelling with signs of oxidative stress including the production of mitochondrial superoxide and cellular depletion of GSH. We also observed a dose-dependent accumulation of polyubiquitinated proteins and loss of proteasomal function in CuO NP-exposed cells, and we could demonstrate misfolding and mitochondrial translocation of superoxide dismutase 1 (SOD1), a Cu/Zn-dependent enzyme that plays a pivotal role in the defense against oxidative stress. The chelation of copper ions using tetrathiomolybdate (TTM) prevented cell death whereas inhibition of the cellular SOD1 chaperone aggravated toxicity. Moreover, CuO NP-triggered cell death was insensitive to the pan-caspase inhibitor, zVAD-fmk, and to wortmannin, an inhibitor of autophagy, implying that this was a non-apoptotic cell death. ZnO NPs, on the other hand, triggered autophagic cell death.

**Conclusions:**

CuO NPs undergo dissolution in lysosomes leading to copper-dependent macrophage cell death characterized by protein misfolding and proteasomal insufficiency. Specifically, we present novel evidence for Cu-induced SOD1 misfolding which accords with the pronounced oxidative stress observed in CuO NP-exposed macrophages. These results are relevant for our understanding of the consequences of inadvertent human exposure to CuO NPs.

**Supplementary Information:**

The online version contains supplementary material available at 10.1186/s12989-022-00467-w.

## Introduction

Copper-based nanoparticles (NPs) are widely used for a variety of industrial and life science applications [1]. For instance, copper carbonate micro- and nanoparticles have been applied on a large scale as a wood preservative [2]. The widespread use of such materials raises concerns regarding their potential adverse health effects. Previous studies have shown that CuO NPs are highly cytotoxic when compared to other metal oxide NPs [3]. Furthermore, evidence has been provided for a so-called Trojan horse effect whereby NPs are internalized by cells and trafficked to lysosomes where they undergo dissolution [4–6]. In a previous study, we found that CuO NPs elicited significantly more cytotoxicity than CuCl_2_ and we also noted an interplay between the NPs and their surface coating with respect to cytotoxicity [7]. In fact, it is possible to “passivate” CuO NPs through surface modification, and poly(ethylene) glycol (PEG) functionalization has been shown to mitigate the toxicity of CuO NPs both in vitro and in vivo [8, 9].

CuO NPs were also found to cause inflammation following short-term inhalation exposure [10, 11], and short-term oral exposure [12], in both cases using rats as a model, and the latter study showed that the immune system may be especially vulnerable to the Cu ions released from Cu-containing particles, as evidenced by lymphoid cell depletion in spleen and thymus [12].

Several authors have invoked autophagic cell death and/or impairment of autophagic flux in CuO NP-exposed cells [13, 14], possibly linked to the induction of mitochondrial damage [15]. Indeed, suppression of mitophagy (i.e., autophagic degradation of damaged mitochondria) was shown to aggravate CuO NP-induced cytotoxicity, suggesting that mitophagy/autophagy is a cytoprotective mechanism [16]. The latter study also showed that superoxide anions originating from damaged mitochondria are involved in CuO NP-induced cytotoxicity. However, the molecular events leading to cell death upon CuO NP exposure remain poorly understood. Nanomaterials may potentially trigger a variety of different forms of programmed cell death [17]. The toxicity of CuO NPs was suggested to result from apoptosis [18], or from the crosstalk between apoptosis and autophagy [19], while Zhang et al. [14] argued in favor of “apoptosis-like” cell death as the pan-caspase inhibitor, zVAD-fmk, failed to rescue cell death in human umbilical vein endothelial cells exposed to CuO NPs. Indeed, Hufnagel et al. [20] reported that the exposure of A549 lung carcinoma cells to CuO NPs failed to trigger apoptosis as evidenced by flow cytometry; instead, an increase in so-called “late” apoptotic cells (in other words, necrotic cells displaying a loss of plasma membrane integrity) was noted. Furthermore, previous studies have provided evidence for the induction of heat shock proteins in cells exposed to CuO NPs, as shown by proteomics using human monocyte-derived cells [21], or by the upregulation of genes encoding heat shock proteins, as shown in transcriptomics studies using lung cell lines of murine and human origin [22, 23]. There are also important lessons to be learned from studies of non-particulate copper. For instance, copper ion-induced toxicity of primary hepatocytes was shown to be related to mitochondrial production of reactive oxygen species (ROS) [24]. Hosseini et al. [25] showed that copper ions triggered ROS formation in isolated rat liver mitochondria. Other investigators have argued that copper overload induces cell death independently of the oxidizing potential of the metal [26]. The authors suggested, instead, that cell death is driven by the induction of protein misfolding. Consequently, cells were shown to react to copper overload by mounting a heat shock response [26]. Here we evaluated a set of industrially relevant nanomaterials, i.e., CuO, WCCo, amorphous SiO_2_, and multi-walled carbon nanotubes (MWCNTs), using the murine macrophage cell line RAW264.7. This initial screening showed that the CuO NPs were the most cytotoxic materials as determined by the benchmark dose (BMD) approach [27]. We therefore focused on CuO NPs to clarify the mechanism of macrophage cell death triggered by these NPs. CuO NPs caused mitochondrial damage and oxidative stress along with proteasome inhibition and the aggregation of proteins in cells. We could also show that CuO NPs caused misfolding and cellular redistribution of superoxide dismutase 1 (SOD1) [28], thus shedding new light on the cytotoxic effects of CuO NPs.

## Materials and methods

### NP characterization

The following nanomaterials were investigated: copper oxide (CuO) from PlasmaChem, Germany, fine tungsten carbide with cobalt binder (WCCo) (tungsten carbide < 88%; cobalt < 12%), from MBN, Italy, multi-walled carbon nanotubes (MWCNTs: 90%; other additives: 10%), from Nanocyl, Belgium, and silicon dioxide (SiO_2_) from LGC Standards, UK. The following primary particle diameters (including average values) were provided by the manufacturers: 3–35 (12) nm (CuO NPs), 23–1446 (170) nm (WCCo NPs), and 3–27 (11) nm (SiO_2_ NPs), while the diameter and length of the MWCNTs was 4–16 (8) nm and 575–3462 (1543) nm, respectively. Additionally, we studied two nanomaterials from the nanomaterial repository of the Joint Research Centre (JRC) of the European Commission. These NPs, designated NM110 (ZnO) and NM103 (TiO_2_), were dispersed according to established protocols [29]. Prior to cell-based assays, the nanomaterials were tested for endotoxin content using the Endpoint Chromogenic LAL Assay (Lonza, Walkersville, MD) as described previously [30]. All values obtained were below 0.5 EU/mL (data not shown). Stock solutions were prepared by dispersing the NPs in 2% fetal bovine serum (FBS) in dH_2_0 at 1 mg/mL, followed by vortexing for 30 s and sonication in a water bath sonicator (Ultrawave, Qseries, SLS, power 100%—HWU; Branson 2200 Ultrasonic Cleaner, frequency: 47 kHz) for 16 min. MWCNTs were sonicated for 45 min by water bath sonication with vortexing every 15 min. Then, samples were diluted to the required working concentrations in cell culture medium as detailed below and vortexed just before use. Hydrodynamic diameter by dynamic light scattering (DLS) was determined for CuO, ZnO, and TiO_2_ NPs suspended at 25 µg/mL in ultrapure Milli-Q^®^ water, Tris–HCl buffer (10 mM), and cell culture medium supplemented with 10% fetal bovine serum (FBS). The average size and polydispersity index (PDI) were analyzed using a Malvern Zetasizer Nano ZS (see Additional file 1: Table S1).

### Macrophage cell culture

The murine macrophage-like cell line RAW264.7 from the European Collection of Authenticated Cell Cultures (Salisbury, UK) was cultured in Dulbecco's modified Eagle's medium (DMEM) supplemented with 10% heat-inactivated FBS (Gibco), 1 mM sodium pyruvate, and 1% penicillin/streptomycin (Gibco). Cells were seeded in 96-well plates (3.5 × 10^4^ cells/well) and incubated for 24 h at 37 °C and 5% CO_2_ prior to experiments. Thereafter, cells were exposed to the indicated concentrations of the test materials (NPs or CuCl_2_ and CoCl_2_ as ionic controls) for 24 h, and BMD_20_ and EC_50_ values were inferred from the dose–response curves as detailed below.

### Cell viability assay

Cell viability was assessed using the Alamar Blue assay, based on the metabolic conversion of resazurin, a non-fluorescent indicator dye, to fluorescent resorufin in living cells (Thermo Fisher Scientific, Sweden). After exposure to NPs, the supernatant was removed and 100 µL of a 1:10 dilution of the Alamar Blue reagent in complete DMEM were added to the cells. Triton-X (0.1%) was used as positive control. As background, culture medium only with 10 µL of Alamar Blue concentrated reagent was used. After 4 h of incubation at 37 °C, the fluorescence was read at 530 nm excitation and 595 nm emission wavelengths using a Tecan Infinite^®^ 200 plate reader (Männedorf, Switzerland). The experiment was performed with at least three biological replicates (shown as red, green, and black symbols) and three technical replicates for each concentration (represented by the small symbols of the same color). Cell viability was quantified by subtracting the background values from each sample and is expressed as the percentage cell viability versus the negative control value, which was set as 100%. Potential interference of test materials with the assay was evaluated in an acellular system by incubating 90 µL of each concentration of the NPs in complete DMEM with 10 µL of Alamar Blue reagent for 4 h at 37 °C, and no interference was observed. The raw data were used for the dose response analysis. Experiments were repeated thrice with technical triplicates for each sample. The dose–response data were used to calculate the benchmark dose response (BMD_20_) (the estimated dose corresponding to 20% of the viability reduction) and the half-maximal effective concentration (EC_50_) (the dose corresponding to a reduction halfway between the lower and upper limits of the dose–response curve) using the PROAST package for R (RIVM, Bilthoven, The Netherlands) as described previously [7]. The BMD is an equipotent dose, meaning that the dose that gives the same effect level compared to control is determined for each test material; this facilitates the comparison of different materials [31]. Cells were preincubated with inhibitors of apoptosis (zVAD-fmk) (20 µM), necroptosis (Nec-1) (30 µM), ferroptosis (Fer-1, 10 µM, and deferoxamine, DFO, 50 µM), and autophagy (wortmannin) (1 µM), as well as the copper chelating agent, TTM (50 µM), and the antioxidants, NAC (2.5 mM) and GSH (0.5 mM) (all from Sigma). All the inhibitors except NAC and GSH were added 30 min before exposure to CuO NPs or CuCl_2_ while NAC and GSH were added 90 min before exposure. Cells were also preincubated with cytochalasin D (10 μg/mL) (Sigma) to study actin-dependent effects on cells. To complement these initial screening studies, additional experiments using ZnO NPs and TiO_2_ NPs (Additional file 1: Table S1) were performed. To this end, RAW264.7 cells were exposed at the indicated concentrations with or without cell death inhibitors and cell viability was determined by the Alamar Blue assay. To understand the role of macrophage migration inhibitory factor (MIF), cells were pre-incubated with 50 µM of 4-iodo-6-phenylpyrimidine (4-IPP) (Sigma-Aldrich) for 1 h. Cells were then exposed to CuO NPs and CuCl_2_ for 24 h. After exposure, the metabolic activity of the cells (indicative of cell viability) was determined by Alamar blue assay.

### Cytokine detection

Cytokine responses to NPs were evaluated using the Luminex^®^ multiplex assay, as previously described by Bhattacharya et al. [30]. Briefly, the supernatant of cells exposed to CuO NPs were collected following exposure for 24 h to concentrations corresponding to BMD_20_ × 0.5, BMD_20_, and BMD_20_ × 2. The samples were collected, centrifuged at 12.000 rpm for 5 min to remove cell debris, and stored at − 80 °C until further analysis. The possible interference of NPs with cytokine detection was also evaluated and no interference was observed. Cytokine profiling was performed for the following cytokines and chemokines: TNF-α, IL-6, IL-1β, IL-10, IL-12, MCP-1 (CCL2), MIP-1β (CCL3), MIP-1α (CCL4), RANTES (CCL5), KC (IL-8, CXCL8). Samples were measured using Bio-Plex 200 system (Luminex^®^ xMAP Technology) operating with Bio-Plex software (Bio-Rad Laboratories, Sundbyberg, Sweden). Cytokine standards were reconstituted in cell culture medium supplemented with 10% FBS. The results are based on three independent experiments each performed with triplicate samples. Results are expressed as pg/mL, based on the standard curves. Finally, the data were analyzed using hierarchical clustering analysis as described previously [30] and a heat map was prepared to visualize the results.

### Autophagy reporter cell assay

The autophagy reporter cell line, RAW-Difluo™ mLC3 expressing the RFP::GFP::LC3 fusion protein was purchased from InVivoGen (Toulouse, France). The cells were handled as previously described [32]. In brief, cells were maintained in DMEM with 10% FBS, 4.5 g/L glucose, 4 mM L-glutamine, 100 U/mL penicillin, 100 μg/mL streptomycin, and 100 µg/mL Zeocin™ (InVivoGen). The cells were tested regularly for mycoplasma using the MycoAlert^®^ mycoplasma detection kit (Lonza). One day prior to each experiment, the reporter cells were seeded on glass coverslips placed in 24 well-plates. Cells were then exposed to CuO NPs or CuCl_2_, or ZnO NPs, at the indicated concentrations for 12 h or 24 h. Then, samples were washed with PBS, fixed with 4% formaldehyde, and counterstained and mounted using Vectashield^®^ Antifade Mounting Medium with DAPI (Vector Laboratories, Burlingame, CA). The cell images were captured using a Zeiss LSM880 confocal microscope operating with ZEN software.

### Transmission electron microscopy

Cells exposed to NPs were processed for TEM according to Gupta et al. [33]. To this end, cells were seeded in 6-well plate one day before the experiment. The following day, cells were exposed to NPs at 10 µg/mL for 2 h. Cells were collected and prefixed with 4% glutaraldehyde in 0.1 M sodium phosphate buffer pH 7.4 for 1 h at 4 °C. Following post-fixation in 1% OsO_4_ in 0.1 sodium phosphate buffer for 1 h at 4 °C, the cells were dehydrated using a gradient of ethanol followed by acetone and LX-112 infiltration and finally embedded in LX-112. Ultrathin sections (50–80 nm) were prepared using a Leica EM UC6 microtome, contrasted with uranyl acetate followed by lead citrate, and examined using a Hitachi HT 7700 electron microscope (Hitachi High-Technologies). Digital images were acquired using a 2kx2k Veleta CCD camera (Olympus).

### Isolation of lysosomes

For the isolation of lysosomes, approx. 50 million cells were collected after 2 h of exposure to CuO NPs at the indicated concentrations. Cells were then processed using the lysosome enrichment kit (Thermo Fisher Scientific). Briefly, cell extracts were prepared in lysosome enrichment reagent A by homogenizing the cell pellet using a pre-chilled Dounce tissue grinder on ice. Lysed cells were then transferred into a microcentrifuge tube and lysosome enrichment reagent B was added. Next, supernatants were collected in fresh tubes by centrifugation (500×*g* for 10 min at 4 °C) and proceeded with the density gradient ultracentrifugation at 145,000×*g* for 2 h at 4 °C. The upper lysosome fraction was collected and mixed with PBS. Next, the diluted lysosome fraction was centrifuged at 18,000×*g* for 30 min at 4 °C and the pelleted lysosomes were stored at − 20 °C until further use. Lysosomal integrity in isolated samples was determined by measuring acid phosphatase activity using the acid phosphatase assay kit (Sigma) and the results confirmed that the isolated lysosomes remained intact (data not shown).

### Dissolution assessment

RAW264.7 cells were seeded at 1 × 10^6^ cells/well in a 6-well plate, one day before each experiment, and were exposed to CuO NPs and CuCl_2_ for 2 h or 24 h at a final concentration of 10 and 20 µg/mL, respectively. Then, samples were collected, and cells were washed with PBS and processed for metal analysis by ICP‐MS [33]. Similarly, isolated lysosomes were collected and processed for ICP-MS. For Cu release in dH_2_O, cell medium, and artificial lysosomal fluid (ALF), NPs were freshly dispersed at 25 µg/mL and incubated for the indicated time-points at room temperature. The simulated fluid was prepared according to published protocols [34] and adjusted to pH 4.5. After incubation, samples were centrifuged at 20,000 rpm for 1 h at 0 °C and the supernatants were carefully collected. Non‐centrifuged dispersions were collected as reference samples to measure the total amount of Cu added. The sample digestion was performed in 32% HNO_3_ for 48 h to ensure complete mineralization. Prior to analysis, samples were diluted to reach approx. 2% of HNO_3_. Cu isotopes were quantified using an iCAP Q ICP‐MS (Thermo Scientific) instrument in KED mode. Calibration standards of 0.1, 1, 10, and 100 μg/L Cu were prepared using a 1000 mg/L reference standard (Spectrascan). Each sample was spiked with a known amount of bismuth as an internal standard with a range of recovery between 80 and 100%. Each sample was injected at least three times and the RSD acceptance was set at 15%. Cell uptake results were normalized according to cell number and expressed as pg Cu/cell. Cu ion release is reported in relation to the concentration in the reference samples.

### Mitochondrial and lysosomal assays

To determine the dissipation of the mitochondrial membrane potential (MMP), RAW264.7 cells were seeded in 6‐well plates and exposed to CuO NPs and CuCl_2_ at the indicated concentrations for 6 h. Some cells were preincubated for 30 min with TTM (50 µM). After exposure to the test materials, cells were incubated with the cell-permeable, fluorescent probe, tetramethylrhodamine, ethyl ester (TMRE) (25 nM) (Abcam, Cambridge, UK) for 30 min at 37 °C in the dark. Next, loss of MMP was validated by imaging of the cells using confocal microscopy, and by flow cytometry. For imaging, cells were seeded on coverslips in 24‐well plates and exposed to CuO NPs and CuCl_2_ for 6 h. The mitochondrial uncoupling agent, CCCP (carbonyl cyanide m‐chlorophenyl hydrazine) (Sigma) (50 µM) was used as a positive control. After exposure, cells were washed and incubated with TMRE (25 nM). Cells were then fixed in 4% paraformaldehyde at room temperature for 15 min followed by washing with PBS. The coverslips were mounted on glass slides using Vectashield^®^ Antifade Mounting Medium with DAPI (Vector Laboratories). Images were captured using a Zeiss LSM880 confocal microscope. The production of superoxide in mitochondria was quantified by flow cytometry using the mitoSOX™ assay (Invitrogen). Briefly, cells were exposed in DMEM supplemented with 10% FBS at the indicated concentrations of CuO NPs and CuCl_2_ for 6 h. After exposure, cells were washed and then stained with the mitoSOX™ dye for 30 min. After staining, cells were washed to remove excess dye. The red fluorescence intensity of 10.000 cells was measured with a BD LSRFortessa™ flow cytometer operating with BD FACS DIVA™ software (BD Biosciences). Data were analyzed and plotted using FCS Express 4 Flow Cytometry software. Furthermore, staining of cells using the pH-sensitive dye (pK_a_ ~ 5.2), LysoSensor™ Green DND-189 (Invitrogen) was performed according to the manufacturer’s instructions. LysoSensor™ Green (1 µM) was added directly to the cell medium and cells exposed or not to CuO NPs were incubated for 1 h at 37 °C. Thereafter, cells were imaged using a Zeiss LSM880 confocal microscope. TMRE-labelled and LysoSensor™ Green-labelled RAW264.7 cells (10.000 events per sample) were also analyzed using the BD LSR Fortessa flow cytometer, as described above.

### Glutathione (GSH) content

GSH levels were determined as described previously [33]. In brief, RAW264.7 cells were seeded in a 96-well plate at a density of 50,000 cells/well one day before each experiment. Next, the cells were exposed to the indicated concentrations of CuO NPs and CuCl_2_ for 12 h. After exposure, the supernatant was discarded, and samples were analyzed by using the GSH-Glow™ assay (Promega) according to the manufacturer’s instructions. The luminescence was measured using a Tecan Infinite^®^ 200 plate reader (Männedorf, Switzerland). For some experiments, cells were preincubated with NAC (2.5 mM) or GSH (0.5 mM) (Sigma) prior to exposure to CuO NPs and CuCl_2_ for 24 h, and cytotoxicity was evaluated using the Alamar blue assay. Additionally, cells were preincubated with or without 50 µM L-buthionine-sulfoximine (BSO) (Sigma-Aldrich) for 2 h and then exposed to the indicated concentrations of CuO NPs or CuCl_2_ for 12 h. After exposure, the samples were analyzed using the GSH-Glow™ assay as above.

### Caspase-3-like activity

RAW264.7 cells were seeded overnight at a density of 1 × 10^6^ cells/well in a 6‐well plate. The next day, cells were exposed to the indicated concentrations of CuO NPs or CuCl_2_ for 6 h. In parallel, cells exposed to etoposide (10 µM) (Sigma) as a positive control. Then, cells were harvested and processed for caspase assay using Vybrant™ FAM Caspase-3 and -7 Assay Kit (Invitrogen, Thermo Fisher Scientific) according to the manufacturer’s instructions. Briefly, cells were incubated with FLICA™ reagent and Hoechst in PBS at 37 °C for 1 h, protected from light. After incubation, the cells were washed twice in 1X apoptosis wash buffer. Next, cells were resuspended in apoptosis wash buffer supplemented with propidium iodide (PI) provided with the kit and immediately analyzed using the NucleoCounter^®^ NC-3000™ (ChemoMetec, Allerød, Denmark). The data were analyzed and plotted using NucleoView™ software (ChemoMetec).

### Western blot analysis

For western blot, 1 × 10^6^ cells were seeded and exposed to CuO NPs and CuCl_2_ as indicated. For some experiments, cells were preincubated with TTM (50 µM). Following exposure, cells were collected and lysed overnight at 4 °C in RIPA buffer [50 mM Tris HCl (pH 7.4), 150 mM NaCl, 1% Triton X-100, 0.25% sodium deoxycholate, 0.1% SDS, 1 mM EDTA]. Protease- and phosphatase inhibitors (Mini EDTA-free Protease Inhibitor Cocktail, Sigma Aldrich; 1 mM PMSF, Thermo Fisher; PhosSTOP™, Sigma Aldrich) and 1 mM DTT (Sigma Aldrich) were freshly added to the buffer. For non-reducing gels, DTT was excluded from the lysis buffer. Cell lysates were centrifuged at 13.000 × g for 15 min and supernatants were collected. The protein concentration was measured using the Bradford assay and 30 or 50 µg were loaded into each well of a NuPAGE 4–12% Bis–Tris gradient gel (Thermo Fisher). Following electrophoretic separation, the proteins were transferred to a Hybond Low-fluorescent 0.2 µm PVDF membrane (Amersham), blocked for 1 h in Odyssey^®^ Blocking Buffer (PBS) (LI-COR), and stained overnight at 4 °C with primary antibodies against HO-1, GPX4, MIF, and SOD1 (all from Abcam), and ubiquitin (Invitrogen). Antibodies to GAPDH (Thermo Fisher Scientific) or β-actin (Sigma Aldrich) were used to control for equal loading, and the goat anti-mouse IRDye 680RD antibody (LI-COR Biotechnology, Germany) was used as secondary antibody. Proteins were detected using the LI-COR Odyssey^®^ CLx scanner operating with the Odyssey^®^ Image Studio software.

### Circular dichroism spectroscopy

To detect structural changes in proteins, we applied CD spectroscopy. To this end, CuO NPs (1, 5, 10, 25, and 50 µg/mL) in dH_2_O or ALF versus CuCl_2_ (10 µg/mL) were incubated with bovine serum albumin (BSA) (20 µg/mL) (purity ≥ 95%) (Calbiochem) in Tris–HCl buffer (10 mM, pH 7.5) for 1 h. CD spectra were then acquired in the far UV spectral region (190–260) using a CD spectrophotometer (J-810 spectropolarimeter, Jasco) with the sample chamber maintained at 25 °C. Measurements were made using a 0.5 mm path length quartz cell. The bandwidth was set to 2 nm, and the integration time was a function of the photomultiplier tube voltage. Buffer alone without protein or NPs or copper salt was used as a blank and BSA alone was included as a further negative control. Additionally, CD spectroscopy was performed using full length recombinant human SOD1 protein (purity > 98%) from Abcam (Sweden) (cat. no. ab112193). The protein was obtained in lyophilized form and reconstituted in endotoxin free distilled water at 0.5 mg/mL. For CD measurements, the stock solution was further diluted in Tris–HCl (10 mM, pH7.2) buffer to achieve a final concentration (15 µg/mL) used in the study. CuO NPs (25 µg/mL) in water or ALF (see “dissolution assessment”) were incubated with recombinant SOD1 (15 µg/mL) in Tris–HCl buffer for 1 h. CD spectra were acquired on far UV-spectral region (190–260) using the CD spectrophotometer (J-810 spectropolarimeter, Jasco).

### Confocal analysis of SOD1

RAW264.7 macrophages were seeded overnight on glass coverslips in a 24‐well plate. The next day, cells were exposed to the indicated concentrations of CuO NPs or CuCl_2_ for 6 h. Some cells were preincubated with 4-IPP (50 µM) (Sigma-Aldrich) for 1 h prior to exposing to CuO NPs or CuCl_2_. After exposure, coverslips were washed twice with 1X PBS and incubated with MitoTracker™ Deep Red (Thermo Fisher Scientific) for 15 min at 37 °C in CO_2_ incubator. Cells were washed again with 1X PBS and fixed with 4% formaldehyde for 15 min at room temperature. Next, the cells were permeabilized in 0.1% of Triton‐X 100 (Sigma‐Aldrich) for 15 min, followed by blocking with 10% of goat serum (Abcam) supplemented with 0.1% of Triton‐X100 for 1 h. Cells were then incubated overnight at 4 °C with rabbit monoclonal anti-SOD1 antibody (1:200, Abcam) prepared in the antibody buffer (8% of goat serum and 0.1% of Triton‐X‐100). The following day, the cells were rinsed in PBS and incubated with Alexa Fluor^®^ 488 conjugated goat anti‐rabbit antibody (1:500, Life Technologies, Thermo Fisher Scientific) for 1 h. Coverslips were then mounted with Prolong Gold antifade reagent with DAPI (Invitrogen) and imaged using a Zeiss LSM900-Airy confocal laser scanning microscope and ZEN software (Zeiss).

### Proteasome activity assay

Proteasome activity in RAW264.7 cells was determined using the fluorometric proteasome 20S assay kit (Sigma). Briefly, 80.000 cells/well was seeded in a 96-well plate. The following day, cells were exposed to CuO NPs or CuCl_2_ for 24 h, while bortezomib (5 nM) was included as a positive control (Sigma). For some experiments, cells were preincubated with TTM (50 µM). Cell medium alone or with CuO NPs or CuCl_2_ without cells were included as blank and as negative controls. Additionally, experiments were performed in which cells were exposed to ZnO NPs (NM110) or TiO_2_ NPs (NM103) for 12 h and 24 h. Following these exposures, the proteasome substrate LLVY-R110 was added, and samples were incubated for 2 h at 37 °C. Fluorescence was recorded using a Tecan Infinite^®^ 200 plate reader (Männedorf, Switzerland).

### Protein aggregation assay

RAW264.7 macrophages were plated on coverslips in a 24-well plate at a density of 1 × 10^6^ cells/mL. Cells were allowed to attach overnight and then were exposed to CuO NPs, CuCl_2_, ZnO NPs, and TiO_2_ NPs for 6 h at indicated concentrations at 37 °C. After exposure, medium was removed, and coverslips were washed twice with 1X PBS. Next, cells were fixed with 4% formaldehyde solution and permeabilized (0.5% Triton X-100, 3 mm EDTA, pH 8.0). Cells were washed again with 1X PBS (Gibco) and then stained with PROTEOSTAT^®^ Aggresome Detection Reagent (1:1000) (Enzo Life Sciences, Lausen, Switzerland) for 30 min at room temperature [35]. After staining, the cells were washed twice with 1X PBS and coverslips were mounted on glass slides with Prolong Gold antifade reagent with DAPI (Invitrogen). Fluorescence was visualized using a Zeiss LSM880 confocal microscope and ZEN software (Zeiss).

### Statistical analysis

The results shown are derived from at least three independent experiments. Data are presented as mean values ± S.D. GraphPad Prism 5 (GraphPad) was used for the statistical analysis. One‐way ANOVA followed by Dunnett's or Tukey's post hoc analysis was applied as indicated. The p values that were considered significant were *p < 0.05, **p < 0.01, ***p < 0.001, and ****p < 0.0001. The PROAST package for R was used for the BMD modeling (refer to “Cell viability assay” section).

## Results

### Cytotoxicity screening of a panel of nanomaterials

**Fig. 1:**
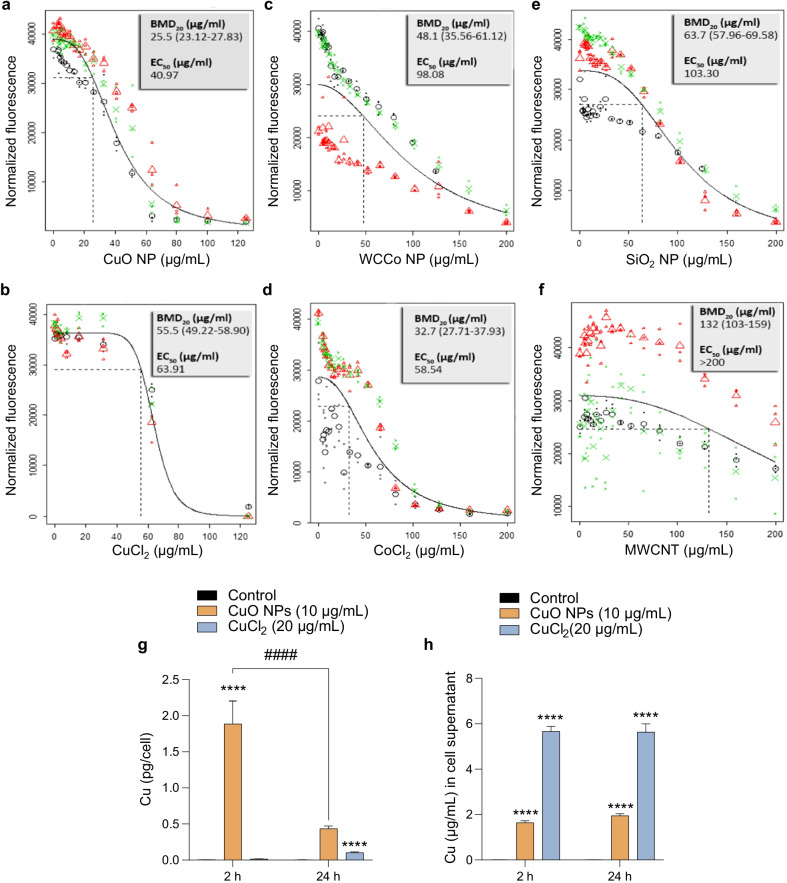
Cytotoxicity screening of a panel of nanomaterials. Cell viability of RAW264.7 cells exposed for 24 h to CuO and WCCo NPs and the corresponding salt (CuCl_2_ and CoCl_2_), or SiO_2_ NPs and multi-walled carbon nanotubes (MWCNTs), was determined using the Alamar Blue assay (metabolic capacity). The benchmark dose (BMD) response for **a** CuO NPs, **b** CuCl_2_, **c** WCCo NPs, **d** CoCl_2_, **e** SiO_2_ NPs, and **f** MWCNTs is reported. The experiment was performed in at least three biological replicates (indicated as red, green, and black symbols). The large symbols in each panel represent geometric mean values of the individual responses, depicted as small symbols. The dashed line in each graph denotes the BMD_20_ dose estimate. BMD_20_ and EC_50_ values are shown in the upper right corner of each panel. **g**, **h** Cu content was quantified by ICP-MS after exposure of cells for 2 h and 24 h to CuO NPs and CuCl_2_. The intracellular concentration of Cu (**g**), and the concentration of Cu in cell supernatants (**h**) is reported. The data are shown as mean values ± S.D. (n = 3). ****p < 0.0001; ####p < 0.001

Using a murine macrophage cell line, we screened the following nanomaterials: CuO, WCCo, and SiO_2_ NPs, and MWCNTs. The materials were selected on the basis of their industrial relevance in the frame of the EU-funded FP7-SUN project [36]. Cytotoxicity towards the RAW264.7 cell line was determined using the BMD approach [31] to facilitate the comparison of the different materials. We observed a high toxicity (defined as BMD_20_ < 100 μg/mL) for CuO, WCCo, and SiO_2_ NPs and a low toxicity (BMD_20_ > 100 μg/mL) for the MWCNTs (Fig. 1a–f). The most toxic material was the CuO NPs (BMD_20_ = 25.50 μg/mL and EC_50_ = 40.97 μg/mL). Overall, the toxicity of the materials tested could be ranked as followed based on their BMD_20_ values: CuO > WCCo > SiO_2_ > MWCNTs. These findings are largely concordant with previous studies using the A549 lung cell line [3]. The toxicity of CuO NPs is commonly attributed to the dissolution of the NPs with the release of copper ions [7, 10]. Indeed, we observed a time-dependent dissolution of CuO NPs in cell culture medium supplemented with serum, but not in dH_2_O (Additional file 1: Fig. S1a). Therefore, we tested the ionic form of Cu and found that CuCl_2_ was less cytotoxic when compared to CuO NPs (Fig. 1a, b) (BMD_20_ = 55.50 μg/mL and EC_50_ = 63.91 μg/mL). To further address whether the released Cu fraction in the cell culture medium contributed towards the observed cytotoxicity, we compared the effect of CuO NPs added directly to cells versus the released Cu fraction of the same dose of CuO NPs dispersed and (partially) dissolved in cell medium for 24 h prior to adding the samples to cells (Additional file 1: Fig. S2a, b). We could confirm the dose-dependent toxicity of the CuO NPs whereas the released Cu fraction did not trigger any effects. We also tested cytochalasin D, an inhibitor of actin polymerization, and observed a significant albeit incomplete rescue from the CuO NP-triggered cytotoxicity (Additional file 1: Fig. S2c).

**Fig. 2:**
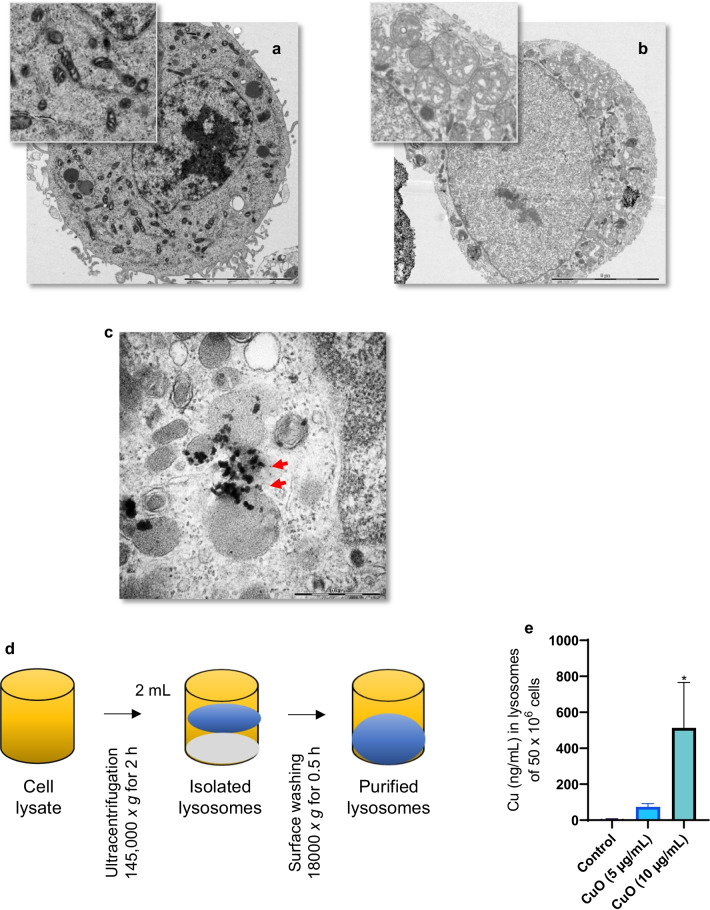
Cellular uptake and lysosomal deposition of CuO NPs. TEM micrographs of control cells (**a**) and cells exposed for 2 h to CuO NPs (10 µg/mL) (**b**). The magnified section in (**b**) shows severely swollen mitochondria in exposed cells compared to control (**a**). The scale bar in (**a**) and (**b**) is 5 µm. **c** TEM micrograph of cells at 2 h of exposure to CuO NPs shows lysosomes as vesicles displaying characteristic multilamellar whirls while NPs are seen as dark/black specks (red arrows). The scale bar in (**c**) is 500 nm. **d**, **e** Dissolution of CuO NPs in ALF (pH 4.5) at the indicated time points was determined by ICP-MS. **d** Purification workflow of lysosomes from CuO NP exposed RAW264.7 macrophages. **e** Concentration of Cu in lysosomes purified from cells after exposure to CuO NPs. Data are mean values ± S.D. (n = 3). *p < 0.05

To determine the intracellular dose of Cu, we performed ICP-MS analysis of cells exposed to CuO NPs (10 µg/mL) or CuCl_2_ (20 µg/mL, the equivalent amount of Cu) (Fig. 1g). Cellular Cu content was significantly higher after exposure to CuO NPs when compared to the ionic form of Cu (at 2 h), but the cellular Cu load was decreased at 24 h. The corresponding results for the cell supernatants are shown in Fig. 1h. We observed a similar pattern of rapid cellular uptake of CuO NPs with a subsequent decrease in cellular copper content at 24 h in a previous study [7]. This could potentially be explained by the exocytosis of Cu as suggested previously [15]. Finally, using TEM, we observed that CuO NPs (10 µg/mL) triggered marked mitochondrial swelling at 2 h of exposure, but no evidence of (apoptotic) chromatin condensation (Fig. 2a, b).

### Cytokine profiling of CuO NP-exposed macrophages

Next, inflammatory responses to CuO NPs were analyzed at three different concentrations corresponding to the BMD_20_ × 0.5, BMD_20_, and BMD_20_ × 2 values obtained at 24 h. Quantification of a panel of murine pro- and anti-inflammatory mediators was performed using a multiplex-based assay, and we performed hierarchical clustering analysis to draw association dendrograms between cytokine responses [37]. LPS was included as a positive control for inflammation. The response to the NPs were less pronounced when compared to LPS, and no IL-1β production was observed in NP-exposed cells (Additional file 1: Fig. S3). Moreover, no effects were seen with respect to TNF-α, another important pro-inflammatory cytokine produced by macrophages. However, the secretion of anti-inflammatory IL-10 as well as IL-12 was suppressed (Additional file 1: Fig. S3). Overall, despite the cellular uptake of CuO NPs, no pronounced inflammatory effects were noted.

### Endo-lysosomal uptake and dissolution of CuO NPs

**Fig. 3:**
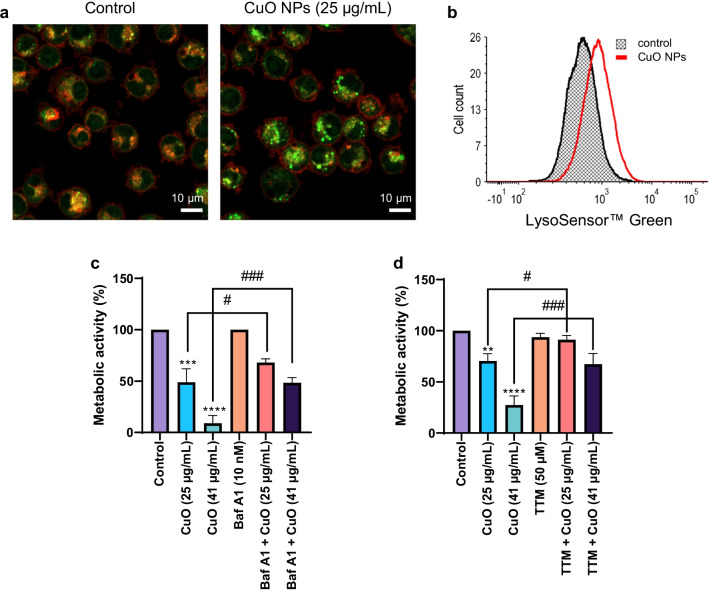
CuO NPs trigger lysosomal acidification. **a** RAW264.7 macrophages were labeled using LysoSensor™ Green following exposure to CuO NPs (25 µg/mL) for 6 h and visualized by confocal microscopy. Scale bars: 10 µm. **b** LysoSensor™ Green fluorescence in control cells and cells exposed to CuO NPs (25 µg/mL) was determined by flow cytometry. Preincubation with bafilomycin A1 (10 nM) (**c**) and TTM (50 µM) (**d**) protected the RAW264.7 macrophages from CuO NP-triggered cell death as shown using the Alamar blue assay. Data are mean values ± S.D. (n = 3). **p < 0.01; ***p < 0.001; ****p < 0.0001 (significant difference between control and treatments); ^#^p < 0.05; ^###^p < 0.001 (significant difference between treatments)

As the CuO NPs were found to be the most cytotoxic of the materials tested, and the underlying mechanism of cell death remains undisclosed, we focused our efforts on these NPs. Endocytosis is a key step in the internalization of NPs [38] and CuO NPs are thought to undergo dissolution in lysosomes following uptake via the endo-lysosomal route [14]. However, few if any studies have determined the actual lysosomal content of copper following exposure of cells to CuO NPs. Therefore, we decided to isolate lysosomes to quantify the copper content in these organelles. TEM imaging suggested that CuO NPs were, indeed, internalized by, or located in close apposition to, lysosomes (electron-dense organelles) (Fig. 2c). We isolated lysosomes by ultracentrifugation (Fig. 2d) and determined the concentration of copper using ICP-MS. We observed a dose-dependent increase in lysosomal copper concentration after 2 h of exposure of RAW264.7 macrophages to CuO NPs (Fig. 2e) confirming that lysosomes are a primary cellular destination for these NPs. The latter result thus provides the intracellular dose at the organelle level. We then investigated whether simulated lysosomal conditions would trigger the dissolution of CuO NPs. To this end, CuO NPs were immersed in ALF (pH 4.5) at 25 µg/mL and incubated up to 6 h. We noted a near-complete dissolution of the NPs as evidenced by ICP-MS (Additional file 1: Fig. S1b). We then checked whether exposure of the RAW264.7 macrophages to the CuO NPs caused lysosomal acidification. Using the pH sensitive dye, LysoSensor™ Green, we clearly observed lysosomal acidification following 6 h of exposure, as shown by confocal microscopy and flow cytometry (Fig. 3a, b). To assess whether lysosomal dissolution plays a role in CuO NP-induced cell death, we preincubated RAW264.7 cells with bafilomycin A1, a vacuolar-type ATPase inhibitor. Cells were significantly protected from CuO NP-triggered cell death (both at the BMD_20_ and EC_50_ dose) (Fig. 3c). Furthermore, preincubating the cells with the copper chelating agent, TTM, also protected from CuO NP-triggered cell death (Fig. 3d). On the other hand, the cathepsin B inhibitor, Ca-074 Me, failed to prevent cell death triggered by CuO NPs as well as CuCl_2_ (Additional file 1: Fig. S4a, b). These results suggested that CuO NP-triggered cell death transpires through endo-lysosomal uptake with lysosomal dissolution and release of cytotoxic ions, while the release of lysosomal enzymes such as cathepsin B does not play a role.

### CuO NPs failed to trigger autophagy or apoptosis

Autophagy is a process whereby cytoplasmic components and organelles are delivered to lysosomes for digestion. Since previous studies have implicated autophagy in CuO NP-induced cell death [13, 19], we decided to test whether CuO NPs (or CuCl_2_) triggered autophagy. To this end, we used the RAW-Difluo™ mLC3 cell line derived from the RAW264.7 cell line. These cells are engineered to express the RFP::GFP::LC3 fusion protein wherein LC3B (microtubule-associated protein 1 light chain 3 beta) is fused to two fluorescent reporter proteins, RFP (acid-stable) and GFP (acid-sensitive), thus allowing for the measurement of autophagic flux [32]. We first performed a dose–response assessment of CuO NPs and CuCl_2_ using the Alamar blue assay (Additional file 1: Fig. S5a, b). Next, confocal microscopy was performed after 12 h of exposure to CuO NPs (10 and 25 µg/mL) and CuCl_2_ (55 µg/mL), but no signs of autophagic flux were noted at 25 µg/mL of CuO NPs and 55 µg/mL of the copper salt (Additional file 1: Fig. S5c). To test whether autophagy was involved in CuO NP-triggered cell death, we preincubated the non-reporter cells with wortmannin, a phosphatidylinositol 3‐kinase (PI3K) inhibitor. However, wortmannin failed to block cell death induced by CuO NPs and CuCl_2_ at the BMD_20_ dose of each compound (Additional file 1: Fig. S4c, d).

To further probe the mechanism of cell death triggered by CuO NPs, we tested pharmacological inhibitors of different cell death pathways including apoptosis (zVAD-fmk, a pan-caspase inhibitor), necroptosis (Nec-1, an inhibitor of receptor-interacting protein 1 kinase), and ferroptosis (Fer-1, a radical-trapping antioxidant, as well as DFO, an iron chelator). However, none of the tested inhibitors protected against cell death triggered by CuO NPs or CuCl_2_ (Additional file 1: Fig. S6a–d). This is in line with a previous study of CuO NPs using primary human endothelial cells in which a very high dose of zVAD-fmk (50 µM) was ineffective in protecting against cell death [14]. We also tested cells for activation of caspases using the FLICA™ affinity labeling method and found that exposure for 6 h to CuO NPs failed to elicit caspase activation while necrosis (PI staining) was observed (Additional file 1: Fig. S7a). Etoposide was included as a positive control (Additional file 1: Fig. S7b).

### CuO NPs trigger oxidative stress in macrophages

**Fig. 4:**
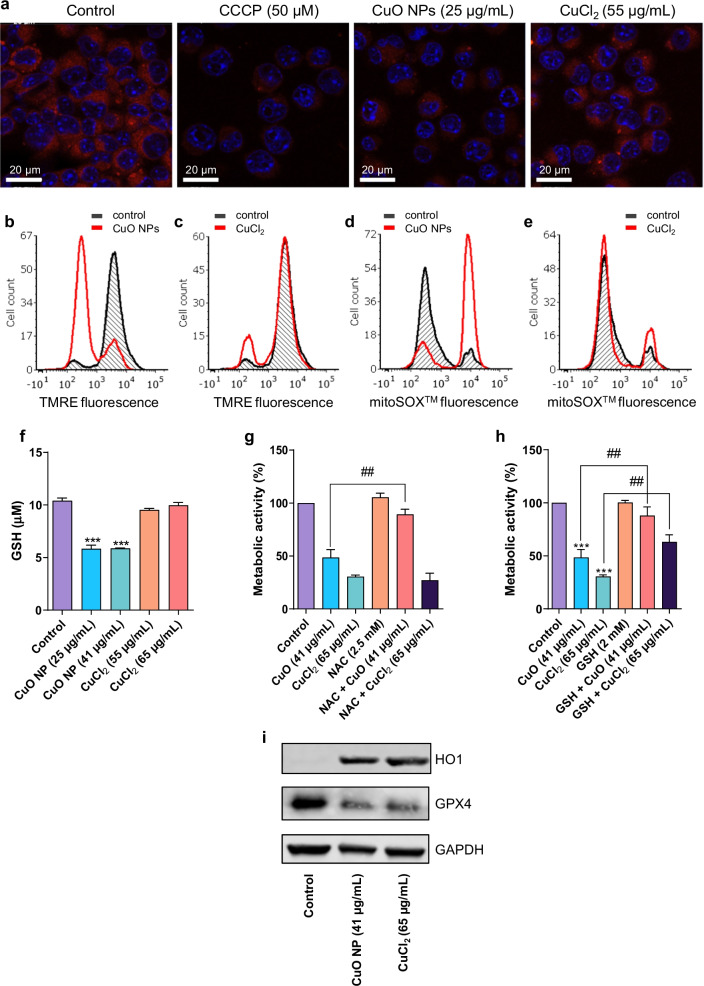
CuO NPs trigger oxidative stress. (a) Mitochondrial membrane potential in RAW264.7 cells was determined by confocal microscopy after 6 h of exposure to CuO NPs and CuCl_2_ as indicated. The uncoupling agent CCCP was used as a control. Confocal images were captured at × 630 magnification. **b**, **c** Mitochondrial membrane potential (TMRE fluorescence) was determined by flow cytometry. Cells were exposed to CuO NPs (25 ug/mL) or CuCl_2_ (50 ug/mL) as indicated. **d**, **e** Mitochondrial superoxide production was determined by flow cytometry using the mitoSOX™ reagent at 6 h of exposure to CuO NPs (25 ug/mL) or CuCl_2_ (50 ug/mL) as indicated. **f** Intracellular reduced glutathione (GSH) content after exposure for 12 h to CuO NPs and CuCl_2_. Supplementation with NAC (**g**) and GSH (**h**) protected cells from cell death triggered by the CuO NPs at 24 h. Data shown are mean values ± S.D. (n = 3). ***p < 0.001, (significant difference between control and treatments); ^##^p < 0.01 (significant difference between treatments). **i** HO-1 and GPX4 expression monitored by western blot after exposure of cells to CuO NPs and CuCl_2_ for 24 h, as indicated. GAPDH was used to control for equal loading

Oxidative stress is frequently invoked in CuO NP-triggered cell death [39, 40] and recent studies have suggested that this effect is mediated by NPs and dissolved ions in tandem [41]. On the other hand, our previous microarray studies did not provide evidence of significant perturbation of oxidative stress-related pathways following pulmonary exposure to CuO NPs [10, 11]. To examine whether CuO NPs induced oxidative stress in macrophages and whether mitochondria were affected, we exposed the RAW264.7 cell line to a BMD_20_ dose of CuO NPs and CuCl_2_ for 6 h. We observed a pronounced loss of the mitochondrial membrane potential (MMP) in cells exposed to CuO NPs (Fig. 4a). The mitochondrial uncoupling agent, CCCP, was used as a positive control. We also monitored TMRE fluorescence by flow cytometry and observed that CuO NPs caused a more pronounced loss of MMP when compared to CuCl_2_ (Fig. 4b, c). Additionally, we applied the fluorogenic dye, mitoSOX™, which permeates live cells where it targets mitochondria and is rapidly oxidized by superoxide, but not by other reactive oxygen species (ROS). Using this reagent, we could show that CuO NPs triggered mitochondrial superoxide production (Fig. 4d), while a less pronounced effect was noted for the CuCl_2_ (Fig. 4e).

Glutathione (GSH) is a major cellular antioxidant, and it protects cells by neutralizing (i.e., reducing) ROS. We queried whether CuO NPs (or CuCl_2_) would affect cellular GSH levels. Indeed, a significant loss of cellular GSH content was observed when RAW264.7 macrophages were exposed for 12 h to CuO NPs, but not in the case of CuCl_2_ exposure (Fig. 4f). Next, we supplemented the cells with NAC (a precursor of GSH) or GSH to determine whether this would rescue the cells from CuO NP-induced cell death. We previously reported that a brief preincubation with NAC was insufficient in preventing CuO NP-induced cell death [7]. However, preincubation for 1.5 h with NAC (Fig. 4g) and GSH (Fig. 4h) provided significant protection when cells were exposed to the EC_50_ dose of the CuO NPs. GSH, but not NAC, also protected from cell death triggered by CuCl_2_ when cells were exposed to the EC_50_ dose. We then asked whether the manipulation of the level of GSH by adding the GSH-depleting agent BSO [42] would sensitize the cells to CuCl_2_. BSO depleted the level of GSH in the RAW264.7 macrophage cell line (Additional file 1: Fig. S8a, b), but BSO alone did not trigger cell death (Additional file 1: Fig. S8c, d). However, preincubation of cells with BSO sensitized the cells to the cell death-inducing effects of CuO NPs (Additional file 1: Fig. S8c). On the other hand, this was evidently not the case for CuCl_2_ (Additional file 1: Fig. S8d). Finally, we also noted a marked induction of heme oxygenase 1 (HO-1), an oxidative stress-induced protein, and a concomitant decrease in the expression of the antioxidant enzyme, glutathione peroxidase 4 (GPX4) following exposure for 24 h to CuO NPs as well as CuCl_2_ (Fig. 4i).

### ZnO NPs trigger autophagy in macrophages

**Fig. 5:**
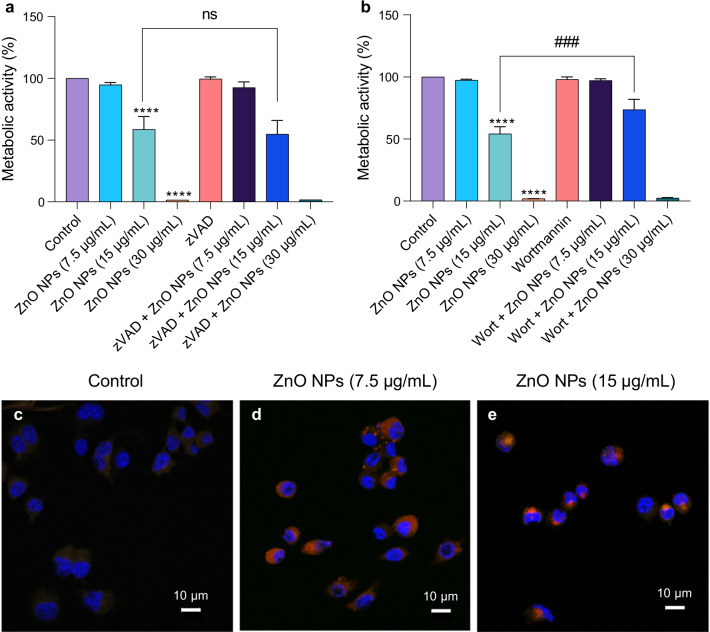
ZnO NPs trigger autophagic cell death. **a** The pan-caspase inhibitor zVAD-fmk failed to protect cells from ZnO NP-induced toxicity. **b** The PI3K inhibitor wortmannin partially protected from ZnO NP-triggered cell death. Cells were preincubated with zVAD-fmk and wortmannin prior to exposure to ZnO NPs. The metabolic activity (indicative of cell viability) was determined using the Alamar blue assay after 24 h. Data shown are mean values ± S.D. (n = 3). ****p < 0.0001, (significant difference between control and treatments); ###p < 0.001 (significant difference between the respective treatments). **c**–**e** Confocal micrographs of RAW-Difluo™ mLC3 cells exposed for 24 h to ZnO NPs. **c** control, **d** ZnO NPs (7.5 µg/mL), and **e** ZnO NPs (15 µg/mL). For results on CuO NPs using the same autophagy assay, refer to Additional file 1

To compare the effects of CuO NPs with those of other metal oxides, we performed additional studies using the “benchmark” materials, NM110 (ZnO NPs) and NM103 (TiO_2_ NPs) obtained from the nanomaterial repository at JRC. The materials were characterized with respect to hydrodynamic diameter and zeta potential (Additional file 1: Table S1). It is notable that all three NPs displayed a similar zeta potential when dispersed in cell culture medium supplemented with 10% FBS. The NPs from JRC were previously subjected to a rigorous in vitro assessment in the EU-funded FP7-MARINA project using 12 cellular models (including the RAW264.7 macrophage cell line) and 10 different cell viability and oxidative stress assays [29]. NM110 was found to be the most cytotoxic and NM103 the least cytotoxic of the tested materials (six in total, i.e., two forms each of the ZnO NPs, SiO_2_ NPs, and TiO_2_ NPs). Here we confirmed that the ZnO NPs were strongly cytotoxic towards the RAW264.7 cell line (Fig. 5a, b). The cells were exposed to the EC_20_ (7.5 µg/mL) and EC_50_ (15 µg/mL) concentrations of ZnO NPs derived from the previous study using the same cell line [29], as well as to 30 µg/mL. To understand the mechanism of cell death, cells were preincubated with zVAD-fmk or wortmannin. The caspase inhibitor zVAD-fmk failed to prevent ZnO NP-induced cell death (Fig. 5a) while wortmannin afforded a partial protection against cell death (Fig. 5b). We also screened inhibitors of other modes of cell death including necroptosis (necrostatin-1) and ferroptosis (ferrostatin-1), as well as the copper-chelating agent, TTM, and the antioxidant, NAC, and found that all the inhibitors, with the exception of NAC, failed to rescue the cells (Additional file 1: Fig. S9a–d). Finally, we applied the ZnO NPs to the RAW-Difluo™ mLC3 cell line, and autophagy was observed at the EC_20_ (7.5 µg/mL) and EC_50_ (15 µg/mL) doses (Fig. 5c–e). Thus, similarly to the CuO NPs, the ZnO NPs triggered caspase-independent cell death. However, unlike the Cu-based NPs, signs of autophagic cell death were noted for the ZnO NPs. The latter observation is in line with several previous studies [43, 44].

### CuO NPs trigger cellular proteostasis collapse

**Fig. 6:**
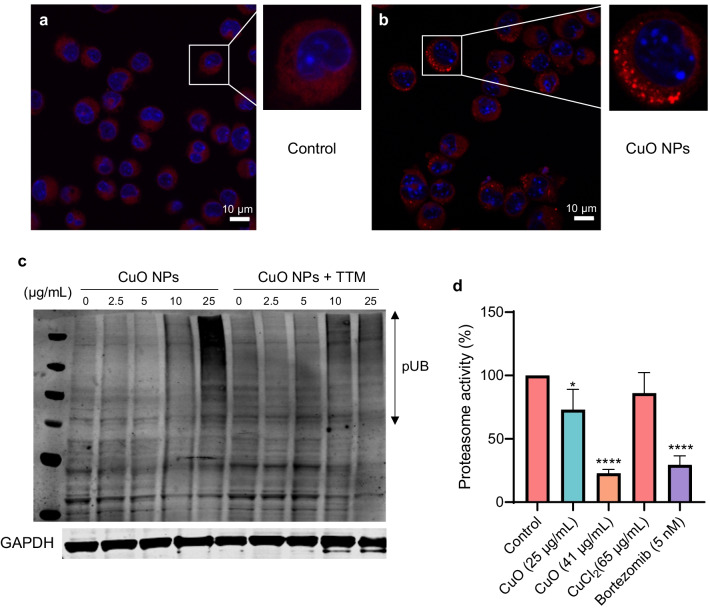
CuO NPs trigger protein aggregation and proteasomal insufficiency. RAW264.7 cells were untreated (**a**) or exposed to CuO NPs (**b**) at 25 µg/mL (BMD_20_) for 6 h and protein aggregation (i.e., aggresome formation due to the aggregation of misfolded proteins) was determined using the PROTEOSTAT^®^ reagent (red). Cell nuclei were counterstained with DAPI (blue). **c** The accumulation of polyubiquitinated proteins after 12 h of exposure of cells to CuO NPs at the indicated concentrations in the presence or absence of TTM (50 µM) was determined using an anti-ubiquitin antibody. GAPDH was used as a loading control. **d** The chymotrypsin-like activity of the 20S proteasome was determined after exposure for 24 h to CuO NPs or CuCl_2_ as indicated. Bortezomib was used as a positive control. Data are shown as mean values ± S.D. (n = 3). *p < 0.05; ****p < 0.0001 (significant difference between control and treatments)

**Fig. 7:**
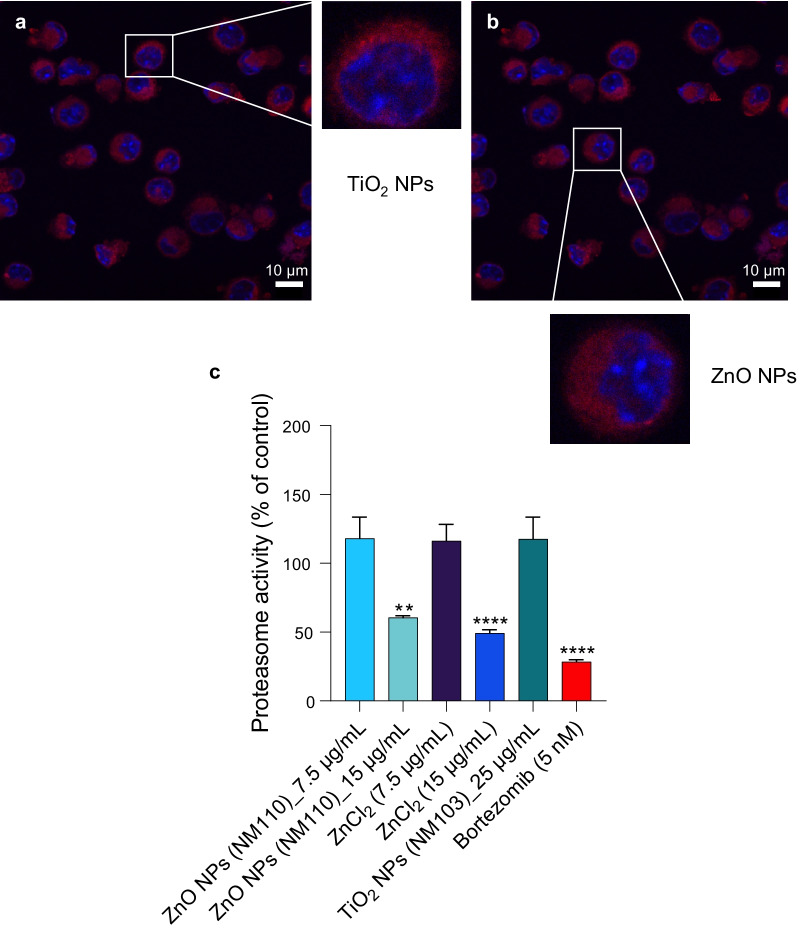
ZnO NPs and ZnCl_2_ inhibit proteasome activity in cells. **a** RAW264.7 cells were exposed for 6 h to TiO_2_ NPs (**c**) or ZnO (**d**) NPs at 15 µg/mL and protein aggregation was determined using the PROTEOSTAT^®^ reagent (red). Cell nuclei were counterstained with DAPI (blue). No signs of protein aggregation were noted. **c** ZnO NPs and ZnCl_2_ triggered inhibition of the 20S proteasome in RAW264.7 cells after 24 h of exposure while TiO_2_ NPs were ineffective in this assay. Bortezomib (5 nM) was included as a positive control. Data shown are mean values ± S.D. (n = 3). **p < 0.01; ****p < 0.0001 (significant difference between control and treatments)

Recent studies suggested that copper ions may drive cell death through protein misfolding [26]. To test whether protein misfolding and/or aggregation occurs in cells subjected to CuO NPs, we applied the PROTEOSTAT^®^ reagent, a dye that emits fluorescence after intercalation into hydrophobic pockets formed by aggregated or misfolded proteins [35]. Indeed, CuO NPs were found to trigger the aggregation of proteins in cells in comparison to control cells (Fig. 6a, b). In contrast, no protein aggregation was observed in cells exposed to TiO_2_ NPs or ZnO NPs (Fig. 7a, b). We also performed western blot for polyubiquitinated proteins as described previously [45], and we could detect an accumulation of polyubiquitinated proteins in cells exposed to a BMD_20_ dose (25 µg/mL) of CuO NPs (Fig. 6c). Preincubation with the copper-chelating agent, TTM prevented the accumulation of polyubiquitinated proteins. The accumulation of polyubiquitinated proteins which are normally destined for degradation by the proteasome [46] suggested that proteasome function might be compromised. Indeed, a dose-dependent loss in activity of the 20S proteasome was observed after 24 h exposure to CuO NPs (Fig. 6d). At the highest tested dose (i.e., the EC_50_ concentration), the effect of the CuO NPs on proteasome activity was comparable to that of the FDA-approved proteasome inhibitor, bortezomib (Fig. 6d). In contrast, we noted that the 20S proteasome activity was enhanced in cells exposed for 12 h to CuO NPs, possibly an early response to counteract the increased burden of misfolded proteins (data not shown). We found that ZnO NPs and ZnCl_2_ administered at the EC_50_ (15 µg/mL) dose also blocked cellular proteasome activity at 24 h, whereas the TiO_2_ NPs failed to do so (Fig. 7c). Overall, these findings show that CuO NPs, which undergo dissolution in lysosomes following cellular uptake, trigger protein misfolding and proteasomal insufficiency in cells.

### CuO NPs trigger misfolding and redistribution of SOD1

**Fig. 8:**
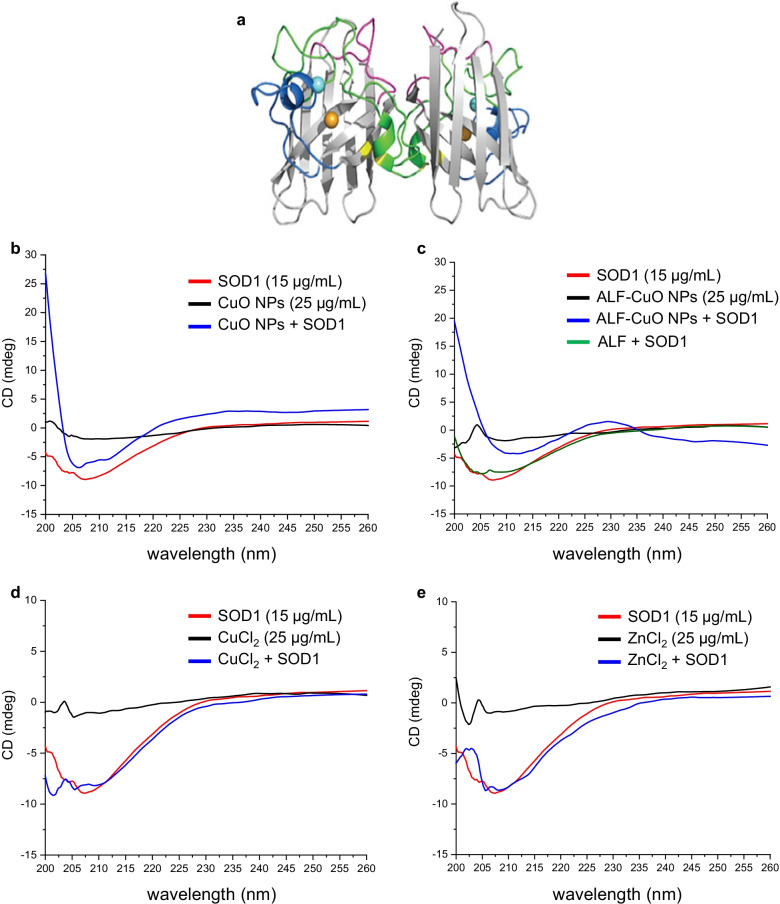
CuO NPs trigger unfolding of SOD1. **a** SOD1 is a dimeric protein comprised of an eight-stranded β-barrel with one Cu (orange) and Zn (cyan) ion bound in each monomer. Reproduced from: Trist BG, Hilton JB, Hare DJ, Crouch PJ, Double KL. Superoxide dismutase 1 in health and disease: how a frontline antioxidant becomes neurotoxic. *Angew Chem Int Ed Engl*. 2021;60(17):9215–46 under a Creative Commons license (CC BY 4.0). **b**–**e** CD spectroscopy measurements were performed to assess conformational changes in recombinant human SOD1. **b** CuO NPs decreased the α-helix and β-sheet conformation of the SOD1 protein. **c** CuO NPs dissolved in ALF (pH 4.5) shifted the α-helix conformation of SOD1 protein from 208 to 212 nm. **d** CuCl_2_ caused only a minor effect on the α-helical content, while **e** ZnCl_2_ showed no effect. ZnO NPs could not be studied due to assay interference

To obtain further information on whether CuO NPs elicit conformational changes in the secondary structure of proteins, we applied CD spectroscopy. BSA was selected as a model protein. Two bands at 208 nm and 222 nm were observed, which are characteristic of the α-helical structure of BSA [47]. The addition of CuO affected the α-helix (208 nm and 222 nm) structures of BSA in a dose-dependent manner (Additional file 1: Fig. S10a) and a similar effect was noted for CuCl_2_ (Additional file 1: Fig. S10b). CuO NPs and CuCl_2_ did not show any interference (Additional file 1: Fig. S10c, d). To address a more relevant intracellular protein, we performed additional CD measurements using SOD1. We selected SOD1 as this protein plays a major role in protecting cells from oxidative stress [28] (Fig. 8a). CuO NPs (25 µg/mL) caused unfolding/misfolding of the SOD1 protein after 1 h of incubation as shown by the decrease in both α-helix and β-sheet conformation (Fig. 8b). CuO NPs dissolved in ALF (pH 4.5) (to mimic the dissolution that takes place in lysosomes) exerted a more significant impact on the α-helix conformation of SOD1 as a shift was noted in the CD spectrum from 208 nm (the α-helical region) to a higher wavelength (212 nm) in addition to the decrease of the amplitude of the peak (Fig. 8c). CuCl_2_ caused only a slight reduction in α-helical content while ZnCl_2_ was ineffective (Fig. 8d, e). However, we could not determine the effects of the ZnO NPs due to a strong interference with CD measurements (data not shown). Taken together, the spectroscopy results suggest an effect of the NPs as well as the dissolved Cu fraction.

**Fig. 9:**
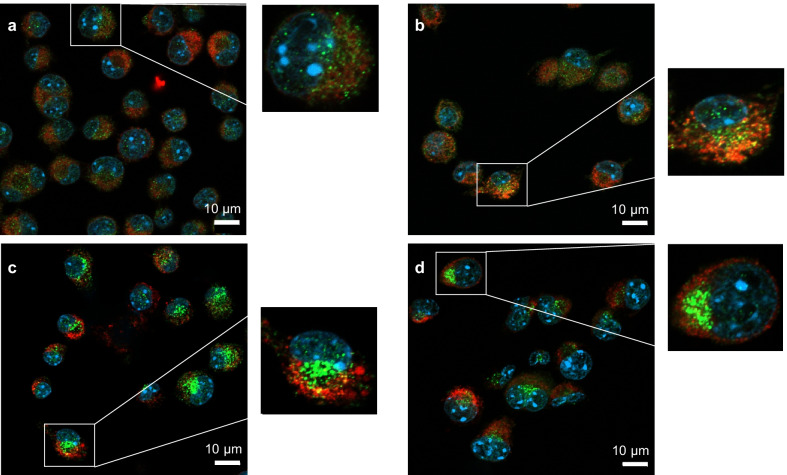
SOD1 redistribution in CuO NP-exposed cells. SOD1 localization in RAW264.7 cells was determined by confocal microscopy. SOD1 was visualized using a specific antibody and an Alexa Fluor^®^ 488-conjugated secondary antibody (green). Mitochondria were detected using MitoTracker™ Red and cell nuclei were counterstained using DAPI (blue). **a** Control; **b** CuO NPs (10 µg/mL); **c** CuO NPs (25 µg/mL); **d** CuCl_2_ (50 µg/mL). The cells were exposed for 6 h

**Fig. 10:**
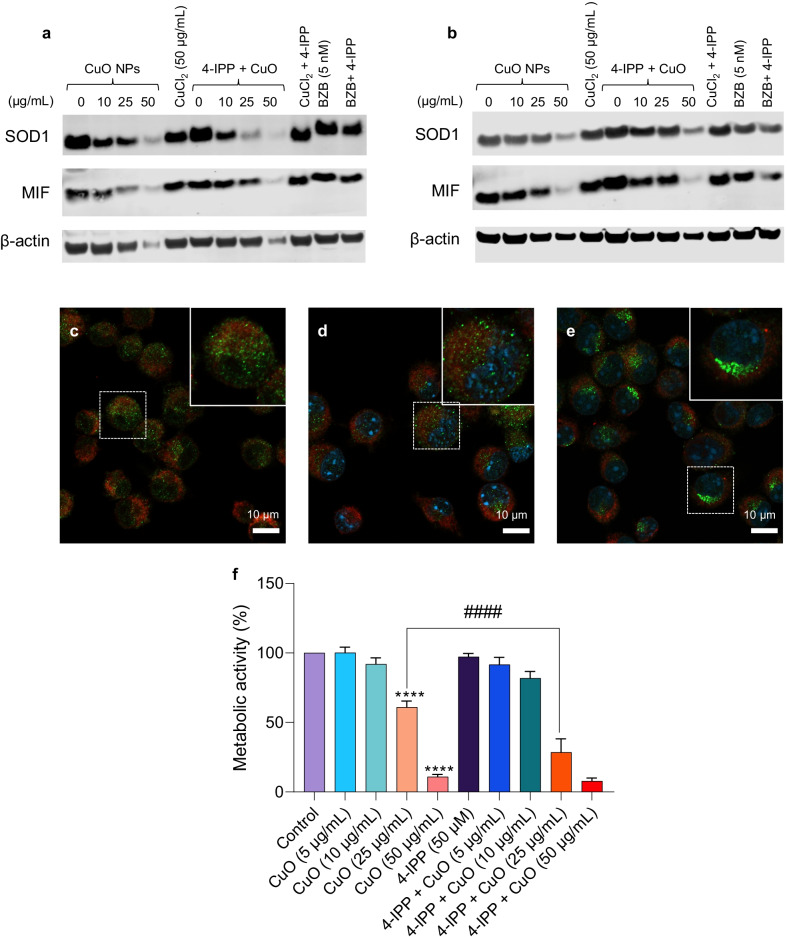
SOD1 expression and role of the SOD1 chaperone, MIF. **a**, **b** Western blot analysis of cells exposed for 12 h to CuO NPs at the indicated concentrations in the presence or absence of the MIF inhibitor, 4-IPP (50 µM). Membranes were probed for SOD1 and MIF, and reprobed using antibodies against β-actin as a loading control. Cells were also exposed to the proteasome inhibitor, bortezomib (5 nM). (**a**) shows the results obtained under non-reducing conditions and (**b**) shows the results under reducing conditions (10 mM DTT added to the lysis buffer prior to the electrophoresis). Note that at high concentrations of CuO NPs (50 µg/mL) the loading control was also affected (under non-reducing conditions) and therefore the loss of the SOD1 monomer at this concentration cannot be evaluated. In cells exposed to CuO NPs under non-reducing conditions, high molecular weight SOD1 oligomers appeared which disappeared when the samples were run under reducing conditions (data not shown). Similar results were obtained in several experiments performed at 12 h or 24 h. **c**–**f** Pharmacological inhibition of the SOD1 chaperone, MIF aggravates CuO NP-induced cell death. RAW264.7 macrophages were exposed for 6 h to medium alone (**c**), 4-IPP (50 µM) (**d**), or 4-IPP plus CuO NPs (10 µg/mL) (**e**) and confocal microscopy was performed to assess the localization of SOD1 (green). Mitochondria and nuclei were identified using MitoTracker™ Red and DAPI (blue), respectively. **f** Cells were exposed for 24 h to CuO NPs at the indicated concentrations in the presence or absence of the MIF inhibitor, 4-IPP (50 µM) and cell viability (metabolic capacity) was determined using the Alamar blue assay. ****p < 0.0001; ^####^p < 0.0001

Previous studies have shown that misfolded SOD1 is deposited onto the cytoplasmic face of the outer mitochondrial membrane in spinal cord neurons [48]. In fact, misfolded mutant SOD1 was found to bind directly to the voltage-dependent anion channel (VDAC1) [49]. We applied confocal microscopy to determine the subcellular localization of SOD1 in RAW264.7 macrophages. The cells were counterstained with MitoTracker™ Deep Red to visualize mitochondria. We found that SOD1 displayed a distributed punctate pattern in unexposed cells (Fig. 9a). In cells exposed to a low dose of CuO NPs (10 µg/mL), SOD1 was co-localized with mitochondria (Fig. 9b). In cells exposed to the BMD_20_ dose of CuO NPs (25 µg/mL), SOD1 remained partially co-localized with mitochondria, but also appeared to aggregate at or near the nuclear membrane (Fig. 9c). Similarly, when cells were exposed to the BMD_20_ dose of CuCl_2_ (50 µg/mL), clear evidence of a juxtanuclear pattern of staining was observed for SOD1 (Fig. 9d). Macrophage migration inhibitory factor (MIF) was previously shown to inhibit SOD1 misfolding in motor neuron-like cells [50]. We decided to apply 4-IPP, a selective inhibitor of the chaperone function of MIF [51], to see whether this would affect the expression or subcellular distribution of SOD1. Using western blot, we observed an apparent loss of SOD1 expression in cells exposed to CuO NPs for 12 h (Fig. 10a). However, previous studies have shown that mutant SOD1 undergoes the formation of disulfide-crosslinked SOD1 oligomers [52]. By adding a reducing reagent, dithiothreitol (DTT) before electrophoresis, the bands corresponding to those oligomers collapsed to a single band corresponding to the SOD1 monomer [52]. Indeed, we found evidence suggestive of high molecular weight oligomers in the absence of DTT, and these bands or smears disappeared under reducing conditions while the expression of the SOD1 monomer was restored (Fig. 10b, and data not shown). In cells preincubated with 4-IPP, the loss of the SOD1 monomer in cells exposed to the BMD_20_ dose of CuO NPs (25 µg/mL) was aggravated, but it was difficult to draw conclusions at higher doses of NPs. Furthermore, confocal microscopy was performed on cells exposed for 6 h to medium alone, 4-IPP (50 µM) alone, or 4-IPP + CuO NPs (10 µg/mL) (Fig. 10c–e). In cells exposed to medium alone or to the inhibitor alone, SOD1 expression was distributed throughout the cell, but in cells exposed to a combination of 4-IPP and a low dose of CuO NPs, a juxtanuclear localization of SOD1 was noted. Finally, in RAW264.7 cells pre-incubated with 4-IPP, the cytotoxicity of CuO NPs was significantly aggravated (at 24 h), while 4-IPP alone had no effect on cell viability (Fig. 10f). Taken together, SOD1, a superoxide-scavenging enzyme, is shown here to undergo Cu-induced misfolding and is redistributed in macrophages that are exposed to a copper overload.

## Discussion

The present study has provided evidence that CuO NPs trigger oxidative stress and cell death in a mouse macrophage cell line with the accumulation of polyubiquitinated proteins and proteasome inhibition. Moreover, we found that CuO NPs dissolved in ALF (pH 4.5) caused the misfolding of recombinant human SOD1 and we observed a redistribution (aggregation) of SOD1 in cells exposed to CuO NPs. Importantly, CuO NPs were deposited in lysosomes and particle dissolution was shown to drive the toxicity as the copper chelating agent TTM protected the cells. On the other hand, inhibitors of apoptosis, necroptosis and ferroptosis failed to rescue cells.

Zhang et al. [53] showed, in a study of 24 metal oxides, that CuO NPs were among the most cytotoxic, as evidenced using the human lung epithelial cell line, BEAS-2B, and the mouse macrophage cell line, RAW264.7. The authors concluded that the most likely mechanism of toxicity was the dissolution of CuO NPs with the release of copper ions and they could show that CuO NPs as well as CuCl_2_ were capable of oxidizing cytochrome c, an essential component of the mitochondrial electron transport chain [53]. CuO NPs have frequently been found to be more cytotoxic than the released copper fraction, and this could be explained by the so-called Trojan horse effect whereby the NPs act as a carrier of ions into the cell [5, 54]. This observation has been confirmed in the present study. Moreover, we can confirm that intracellular dissolution is a key event underlying the toxicity in macrophages as the prevention of the acidification of lysosomes (bafilomycin A1) or chelation of copper ions (TTM) both rescued the cells from CuO NP-triggered cell death. We also showed that supplementation of the cells with NAC or GSH suppressed cell death, arguing in favor of a role of oxidative stress. Indeed, we provided evidence of several indices of oxidative stress including a marked reduction in cellular GSH levels and the induction of HO-1. The induction of oxidative stress with upregulation of HO-1 was also shown in the human hepatoma cell line HepG2 exposed to CuO NPs [55, 56]. Saporito-Magriná et al. [26] proposed that the protective role of GSH in cells subjected to Cu overload is based on the binding of Cu ions, thus preventing the metal interaction with proteins. This is to some degree a rediscovery of the well-known fact that GSH binds Cu especially in conditions of Cu overload. Hence, previous studies have suggested a model whereby Cu is complexed by GSH immediately upon gaining access to the cell and is then transferred to metallothioneins (MTs) [57, 58]. Furthermore, using electron spin resonance spectroscopy, Milne et al. [59] could show that GSH inhibits free radical formation by Cu, and they concluded that the protective effect of GSH may be attributed to the stabilization of Cu in its + 1 oxidation state. GSH may thus act both as a ligand for Cu(I) and as a radical scavenger. Several studies have shown that metal/metal oxide NPs are internalized by cells through endocytosis and are trafficked to lysosomes where they are transformed and release ions [60, 61]. Recent studies using the BEAS-2B cell line [62] or primary human umbilical vein endothelial cells [14] showed that lysosomal deposition of CuO NPs and lysosomal acidification are key processes driving the cytotoxicity of these nanomaterials. Semisch et al. [63] found, using the A549 lung cell line, that copper derived from CuO NPs as well as CuCl_2_ accumulated both in the cytoplasmic and the nuclear compartments/fractions by using ICP-MS. However, the present study is the first to quantify the lysosomal content of Cu following exposure to CuO NPs (as shown by ICP-MS on isolated organelles). Furthermore, we have provided evidence that the exposure of macrophages to CuO NPs leads to a marked accumulation of polyubiquitinated proteins and to proteasome inhibition (discussed below). These findings are in line with a previous report in which the authors observed that copper overload caused protein aggregation and cell death [26]. However, the authors argued that oxidative stress was not required for cell death to occur, which contrasts with the present study, as we have shown that CuO NPs trigger oxidative stress.

There are many examples in the literature demonstrating distinct biological activities of charge-controlled NPs including the elegant study of Xia et al. [64] in which the authors showed that the noncovalent attachment of polyethyleneimine (PEI) to the surface of mesoporous silica particles increased cellular uptake. Using a set of particles coated with 0.6, 1.2, 1.8, 10, and 25 kD polymers, the authors could show that the cytotoxicity potential towards pancreatic cancer cell lines could be adjusted by altering the polymer length. Importantly, cell-specific uptake pathways, and not only the surface charge, may come into play [65, 66]. Moreover, it is also practically unavoidable (for most NPs) that serum proteins or other biomolecules adsorb to the particle surface, and this may, in turn, depend both on particle size and surface charge, as shown in a previous study by Lundqvist et al. [67]. Others have found more recently that the accessibility of amino groups or amine “bulkiness” is more important than the average surface charge for protein corona composition and for the resulting cellular association [68]. Notwithstanding, it is conceivable that the adsorption of biomolecules present in cell culture medium may serve to “equalize” the surface charge of the NPs (as shown here for the CuO, ZnO, and TiO_2_ NPs). We previously investigated a set of CuO NPs with different surface modifications, i.e., anionic sodium citrate and sodium ascorbate, neutral polyvinylpyrrolidone (PVP), and cationic PEI versus uncoated NPs using the RAW264.7 macrophage cell line [7]. Using the BMD approach, we could show that the NPs coated with PEI along with the uncoated NPs were the most cytotoxic, followed by the PVP-coated NPs, whereas the citrate- and ascorbate-coated NPs were the least toxic. However, the different surface modifications only modestly affected Cu dissolution in cell culture medium [7]. Taken together, CuO NP-triggered cytotoxicity is likely driven by the intracellular (lysosomal) dissolution of the particles, though we cannot discount a role of the surface charge of the particles for cellular uptake in our model.

We also performed cytokine profiling of macrophage-like RAW264.7 cells exposed to CuO NPs and found that the secretion of IL-10 and IL-12 was reduced. The suppression of IL-10 production implies that CuO NPs may affect macrophage polarization [69]. However, CuO NPs did not affect TNF-α production significantly. Additionally, we did not detect IL-1β production in the present cell model, thus ruling out pro-inflammatory pyroptosis as the mode of cell death [32]. Indeed, we were unable to rescue the cells using the pan-caspase inhibitor, zVAD-fmk, and we could not detect any ultrastructural features of apoptosis, suggesting a non-apoptotic mode of cell death. Instead, TEM revealed a pronounced swelling of mitochondria in CuO NP-exposed cells which is reminiscent of necrotic cell death. Furthermore, it is noted that caspases are redox-sensitive enzymes, and that excessive oxidative stress may inactivate the apoptotic machinery [70]. It is also notable that the autophagy inhibitor wortmannin failed to protect the cells from CuO NPs while cell death triggered by ZnO NPs was partially reversed. Thus, at least in the present cell model, we may exclude autophagic cell death as well as apoptosis. Instead, the CuO NP-triggered cell death described herein is somewhat reminiscent of the previously identified form of cell death that is referred to as paraptosis. The latter cell death involves extensive endoplasmic reticulum (ER) vacuolization in the absence of caspase activation [71]. Previous studies have shown that proteasomal dysfunction contributes to the ER dilation and ER stress in paraptosis [72], and a link was suggested between copper overload, proteasome inhibition, and cell death with massive vacuolization [73]. In the latter study, the authors surmised that paraptosis may serve as a backup pathway of cell death that is triggered when a critical threshold of unfolded proteins is reached, and the apoptotic machinery is incapacitated [73]. However, we have not observed ER dilation but could show instead that lysosomes and mitochondria are key cellular organelles involved in CuO NP-induced oxidative stress and cell death.

**Fig. 11:**
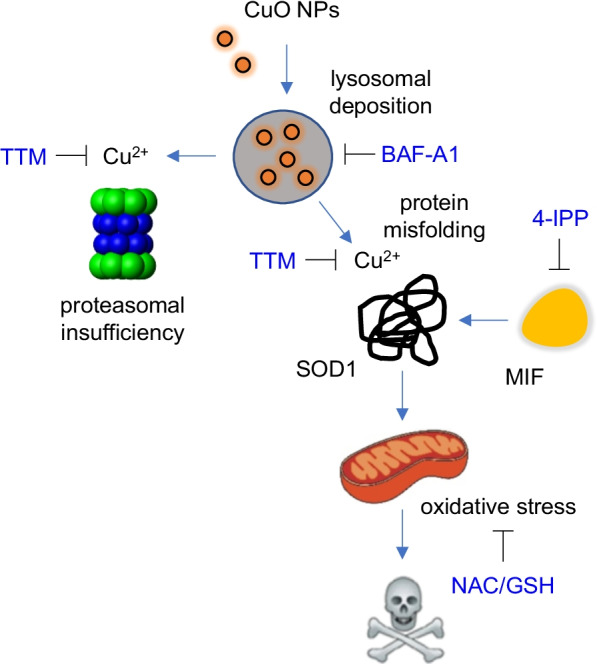
Current model of CuO NP-triggered cell death. The figure depicts cellular uptake of CuO NPs by macrophages leading to their deposition in lysosomes. This is followed by the dissolution of the NPs at acidic pH with subsequent effects of copper ions on the cells leading to protein misfolding and mitochondrial dysfunction with ensuing oxidative stress. Furthermore, we have demonstrated proteasome inhibition in cells exposed to CuO NPs. This could potentially be due to massive protein misfolding leading to proteasomal insufficiency and/or to a direct inhibitory effect of Cu on the proteasome. MIF is an SOD1 chaperone. Consult text for details

The present finding that CuO NPs cause inhibition of the 20S proteasome in cells is novel and it could be explained by direct as well as indirect effects of Cu ions. Hence, one possibility is that the Cu ions caused intracellular protein misfolding leading to proteasomal insufficiency. Our CD spectroscopy analyses using BSA as a model protein, and cellular studies using the aggresome-specific fluorescent dye both support this suggestion. On the other hand, it is conceivable that CuO NPs could also directly suppress the proteasome. Falaschetti et al. [74] reported that metal oxides including Fe_3_O_4_ NPs with varying surface properties were adsorbed to the purified 20S proteasome which could suppress or enhance its activity. However, whether such interactions also took place in intact cells was not investigated [74]. On the other hand, it is well known that copper-binding compounds such as clioquinol and pyrrolidine dithiocarbamate inhibit the proteasome [75]. Additionally, neocuproine, when complexed with CuCl_2_, also blocked the proteasome, and while the organic copper complex triggered ROS formation, this was not required for proteasome inhibition since antioxidants like NAC could not reverse the inhibitory activity of the complex [76]. We showed here that CuO NPs caused accumulation of polyubiquitinated proteins in macrophage-like cells, and this could be due to massive protein misfolding thereby overwhelming the cellular capacity for protein degradation and/or it could be due to inhibition of the proteasome itself, or both (Fig. 11). At any rate, the accumulation of ubiquitin-tagged proteins was mediated by copper ions as this was prevented by TTM. Moreover, CuO NPs dissolved in ALF caused significant changes in the conformation of SOD1, as shown by CD spectroscopy. In fact, the shift in the actual conformation of the protein is a typical signature of aggregated proteins [77]. Conversely, aggregation of proteins may trigger irreversible effects on protein conformation. Therefore, one may argue, based on the present CD measurements, that the dissolution of CuO NPs in ALF (to mimic lysosomal dissolution) imposes irreversible conformational changes in the SOD1 protein. It is noted that the CD spectroscopy measurements were performed in Tris–HCl buffer (pH 7.5), and that the CuO NPs tended to agglomerate in this buffer when compared to cell culture medium (Additional file 1: Table S1). However, CD spectroscopy cannot be performed using serum-containing medium. Therefore, a strict extrapolation between acellular and cellular assays is not possible. Nevertheless, it is relevant to note that we have provided evidence of misfolding of wild-type, not mutant, SOD1 protein. Other investigators suggested that wild-type SOD1 acquires toxic properties (at physiological temperature) when subjected to so-called molecular crowding [78]. However, the protein was demetalated (i.e., Cu and Zn ions were removed) which reduces the stability of the protein. Our findings suggest instead that Cu “overload” may be deleterious for SOD1.

The present observation that CuO NPs caused protein misfolding in cells is in accordance with previous “omics” studies. Tarasova et al. [21] showed that CuO NPs impacted the “unfolded protein response” and “heat shock response” pathways in the THP-1 cell line using LC–MS/MS-based proteomics approaches. Hence, exposure of THP-1 cells for 24 h at 22 µg/mL (i.e., the EC_50_ concentration for this cell line) caused the up- and downregulation of 86 and 91 proteins, respectively. Notably, almost one third (24 out of 86) of the upregulated proteins were related to the “unfolded protein response” and “heat shock response” pathways, including heat shock protein 70 (HSP70) family member 6 (encoded by the *HSPA6* gene) [21]. The HSP70 family members are ubiquitous molecular chaperones that play important roles in protein folding and in protecting cells from various forms of cellular stress [79]. Hanagata et al. [22] performed microarray analyses of the human lung adenocarcinoma cell line A549 exposed to 25 μg/mL CuO NPs for 24 h and found that several HSP-encoding genes were upregulated (along with several other genes). The authors speculated that the upregulation of HSPs was a sign that the CuO NPs had stimulated protein “denaturation” in cells [22]. Boyadzhiev et al. [23] performed microarray analyses on immortalized FE1 mouse lung cells exposed to CuO NPs at a range of doses (1–25 µg/mL). The results showed that “unfolded protein response” was one of the most significantly affected pathways at the highest dose tested. The oxidative stress related *Hmox1* gene was also upregulated. Triboulet et al. [80] performed a proteomics screen of RAW264.7 macrophages using 2D gel electrophoresis and could show that proteins implicated in oxidative stress responses, glutathione biosynthesis, and mitochondrial function (in particular, oxidative phosphorylation complex subunits) were affected. The authors argued that cells responding to copper-based NPs “made a strong effort to increase their glutathione level”. The authors also inferred from the proteomics analysis that the expression of Cu/Zn-dependent SOD1 was diminished while mitochondrial Mn-dependent SOD2 was not affected [80]. Boyles et al. [81], in turn, applied an untargeted metabolomics approach to assess the impact of CuO NPs (10 µg/mL) on A549 cells, and noted that CuO NPs induced regulation of metabolites involved in oxidative stress, i.e., a depletion of the reduced form of glutathione (GSH) and an increase in the oxidized form of glutathione (glutathione disulfide, GSSG), accompanied with an increase in cysteine-glutathione disulfide, the product of GSH oxidation. Kooter et al. [82] compared the toxicity of pristine and surface modified CuO NPs in primary human normal and asthmatic lung cells exposed to CuO NPs (using a dose that triggered a maximum of 30% cell death). Interestingly, *HSPA6* was identified among the 48 differentially expressed genes showing overlap across all the exposures. Hufnagel et al. [20] applied an RT-PCR based array spanning 95 genes and found that *HMOX1* as well as *HSPA1A* (encoding heat shock 70 kDa protein 1, also known as HSP72) were both upregulated in A549 cells exposed to CuO NPs. It also deserves mentioning that we recently performed a transcriptomics study of THP-1 cells exposed to a panel of 31 nanomaterials including pristine CuO NPs at a low dose, corresponding to a maximum of 10–15% cell death (EC_10_) for 24 h [83], and we found that the most upregulated gene in cells exposed to CuO NPs was *HMOX1* encoding HO-1. Furthermore, 10 of the 20 most upregulated genes encoded different MTs. Indeed, it should be noted that several MT-encoding genes were also identified in the studies reported above which is unsurprising as MTs are important metal-binding proteins [84]. We also documented a strong induction of MT-encoding genes in rat lungs following a short-term inhalation exposure to surface coated or pristine CuO NPs (the same uncoated NPs were used in the present study) [10, 11]. Overall, copper-responsive genes/proteins have been found to encompass metal binding proteins (MTs) as well as antioxidant proteins (HO-1); moreover, proteins that respond to unfolded/misfolded proteins (HSPs) should also be considered as part of the canonical copper response. Together with SOD1, MTs have the highest affinity for Cu(I) [85]. However, upregulation of MTs is also seen in cells exposed to ZnO NPs [86]. Using a panel of surface-modified ZnO NPs, we previously showed that the cellular response to ZnO NPs including the induction of MT-encoding genes is largely dependent on particle dissolution [87]. However, ZnO NPs have been found to undergo extensive dissolution in cell culture medium and the extracellular chelation of Zn ions was shown to be sufficient to completely rescue cells from cell death [88]. On the other hand, we observed very low or no dissolution of the CuO NPs in water and a slow dissolution rate in cell culture medium whereas dissolution occurred promptly in ALF. This is in line with previous results showing dissolution of CuO NPs in cell culture medium and low solubility in Milli-Q^®^ water and PBS [89]. The high (but slow) dissolution of CuO NPs in cell medium despite the near-neutral pH has been ascribed to the chelating effect of amino acids and/or proteins [54, 90]. Notwithstanding, the dissolution of CuO NPs is believed to be pH-dependent [90]. Hence, the lysosomal acidic environment can transform CuO NPs into Cu(II), which can be transformed via a Fenton-like reaction into Cu(I) [90]. The latter observation may clarify why TTM is able to effectively block CuO NP-induced cell death but not CuCl_2_-induced cell death in our model (data not shown), as TTM is known to bind Cu(I) with high affinity.

SOD1, a largely cytosolic enzyme, requires Cu to scavenge superoxide [28]. However, under physiological conditions, the level of intracellular free Cu is subject to strict regulation, and soluble Cu chaperones are required to directly transfer Cu to its specific cellular targets. SOD1 is known to obtain Cu through its interaction with the cytosolic protein termed “Cu chaperone for SOD1” (CCS) [91]. CCS-independent pathways also exist, and these appear to depend, at least in part, on GSH [92]. Misfolded SOD1 also interacts with HSPs and with MIF, a chaperone for SOD1 in non-neuronal cells. Interestingly, elevating MIF in neuronal cells was found to suppress accumulation of misfolded SOD1 and its association with mitochondria [93]. In the present model, the macrophage-like cells readily internalized CuO NPs, leading to a cytosolic Cu overload resulting from the dissolution of the NPs in lysosomes. Furthermore, in addition to the unfolding/misfolding of SOD1, leading to the redistribution of SOD1 to mitochondria, the cellular pool of GSH was depleted. Hence, the capacity to “buffer” excess Cu was diminished. While we cannot exclude that the NPs themselves interact with target proteins, it is likely that the Cu ions triggered misfolding of SOD1 through interactions with cysteine (Cys) residues in SOD1. Our CD spectroscopy measurements support this conclusion as CuO NPs caused a reduction in the α-helical content of SOD1, whereas CuO NPs dissolved in ALF triggered a marked shift in the CD spectrum indicative of a conformational change in the native structure of the protein in addition to the strong reduction in α-helical content of SOD1. We speculate that the suppression of proteasome activity and/or induction of proteasomal insufficiency due to massive protein misfolding in CuO NP-exposed cells may further hamper cellular defenses, leading to oxidative stress that remained unchecked due to the loss of SOD1 function. It is worth noting in this context that proteostasis collapse is a hallmark of aging [94]. The accumulation of misfolded and/or aggregated proteins may affect especially postmitotic cells such as neurons. Indeed, loss-of-function mutations in *SOD1* with misfolding of the SOD1 protein are a well-known cause of the neurodegenerative disease amyotrophic lateral sclerosis (ALS) [95]. The present findings, using a non-neuronal cell model, are novel insofar as a cellular copper overload appears to trigger misfolding of wild-type (not mutant) SOD1 with a redistribution of SOD1 aggregates to a juxtanuclear position. The compartmentalization of mutant SOD1 aggregates was previously shown to determine their toxicity [96]. Hence, “toxic” aggregates were shown to localize to the so-called juxtanuclear quality control compartment (JUNQ) which also triggered the sequestration of HSP70, preventing the timely delivery of misfolded proteins to the proteasome [96]. We did not ascertain whether misfolded SOD1 congregated specifically in the JUNQ, but our results showed (i) that SOD1 aggregated in a juxtanuclear position in cells exposed to a high concentration of CuO NP or to a low concentration of CuO NPs combined with 4-IPP, a specific inhibitor of the SOD1 chaperone, MIF [51], and (ii) that CuO NP-exposed cells displayed a generalized protein aggregation, as evidenced using an aggresome-specific dye, along with proteasomal insufficiency. In contrast, protein aggregation was not seen in cells exposed to ZnO NPs, even though 20S proteasome activity was reduced. Taken together, the data suggest that proteasomal insufficiency and the misfolding of SOD1, whether caused by mutations or by a metal imbalance in the cell, are linked. Furthermore, the formation of protein aggregates in cells may not be the actual source of toxicity and might represent an attempt to remove “harmful” proteins from the cytosol. Instead, protein misfolding as seen in the present model of copper overload, or in cells expressing mutant SOD1 [96], may lead to an insurmountable burden on protein control systems.

## Conclusions

The present study has investigated the mechanism of toxicity of CuO NPs using a murine macrophage cell line as a model. We addressed the role of lysosomal deposition and dissolution of the particles, and we revisited the role of oxidative stress in cells exposed to CuO NPs. Our results showed that CuO NPs triggered a non-apoptotic form of cell death characterized by protein misfolding, proteasomal dysfunction, and oxidative stress with marked mitochondrial swelling. Chelation of copper ions with TTM (also used for the treatment of disorders of copper excess) [97] prevented macrophage cell death, and we could confirm the Trojan horse mechanism [98] insofar as NP uptake with subsequent lysosomal dissolution was found to be required for cell death in the current model. We suggest that this Cu-dependent mode of cell death may be referred to as *cuproptosis* to distinguish it from classical, caspase-dependent apoptosis. Indeed, the term cuproptosis was recently introduced to describe a novel form of Cu-dependent cell death that is distinct from apoptosis and ferroptosis in cancer cells that have adapted to proteotoxic stress [99]. The delivery of Cu to intracellular compartments, whether through Cu ionophores [100] or through the cellular uptake of Cu-based NPs, as shown here, could potentially trigger a common form of Cu-dependent cell death. Finally, the fact that CuO NPs caused misfolding and aggregation of SOD1 correlates well with the increased level of mitochondrial superoxide evidenced in CuO NP-exposed cells (Fig. 11), and these results underscore the prominent role for mitochondria-driven oxidative stress in CuO NP-triggered cell death.

## Supplementary Information

**Additional file 1**. Supporting Table S1 and Figures S1-S10.

## Data Availability

The data required for the interpretation of the results are contained in the publication.
