# Supplementary material for: Copper oxide nanoparticles trigger macrophage cell death with misfolding of Cu/Zn superoxide dismutase 1 (SOD1)
Source: Part Fibre Toxicol. 2022 May 10;19:33. doi: 10.1186/s12989-022-00467-w (PMC9088059; doi:10.1186/s12989-022-00467-w)
Supplement: Supplementary file 1 — Additional file 1. Supporting Table S1 and Figures S1-S10. [file 12989_2022_467_MOESM1_ESM.pdf]

Supporting Information

Copper oxide nanoparticles trigger macrophage cell death with misfolding of Cu/Zn superoxide dismutase 1 (SOD1)

G. Gupta[a] F. Cappellini[a,b], L. Farcal[a], R. Gornati[b], G. Bernardini[b], and B. Fadeel[a]

[a]*Institute of Environmental Medicine, Karolinska Institutet, Stockholm, Sweden;* [b]*Department of Biotechnology and Life Sciences, University of Insubria, Varese, Italy.*

Table S1. Nanoparticle characterization.

	Primary size[a] (nm) (min-max) (and/or average)	DLS					
		Z-average (nm)			Zeta potential (mV)		
		Milli-Q® water	[b]*Cell medium*	[c]*Tris-HCl buffer*	*Milli-Q® water*	[b]*Cell medium*	[c]*Tris-HCl buffer*
CuO	3-35 (12)	129 ± 1	152 ± 2	837 ± 44	28 ± 0.5	-10 ± 1	-2.5 ± 0.4
ZnO	158	240 ± 1	59 ± 6	428 ± 11	-6.6 ± 0.2	-8.5 ± 0.2	-15 ± 1
TiO_2	25	166 ± 3	178 ± 1	1661 ± 20	-4 ± 0.1	-11 ± 0.5	-11 ± 0.7

[a]The primary particle sizes were determined by TEM and this information was retrieved from the EU-funded FP7-SUN project (CuO) (Hristozov et al., Nanotoxicology. 2018;12(7):747-65) and the FP7-MARINA project (ZnO [NM110] and TiO_2 [NM103]) (Farcal et al., PLoS-One. 2015;10(5):e0127174). The latter NPs were obtained from the nanomaterial repository at the Joint Research Center (Totaro et al., Regul Toxicol Pharmacol. 2016;81:334-40).

[b]Cell medium: Dulbecco's Modified Eagle Medium (DMEM) supplemented with 10% FBS (used for cell culture).

[c]10 mM Tris-HCl buffer (pH 7.2) used for CD spectroscopy measurements of BSA/SOD1 (refer to Fig. 8 and Fig. S10).

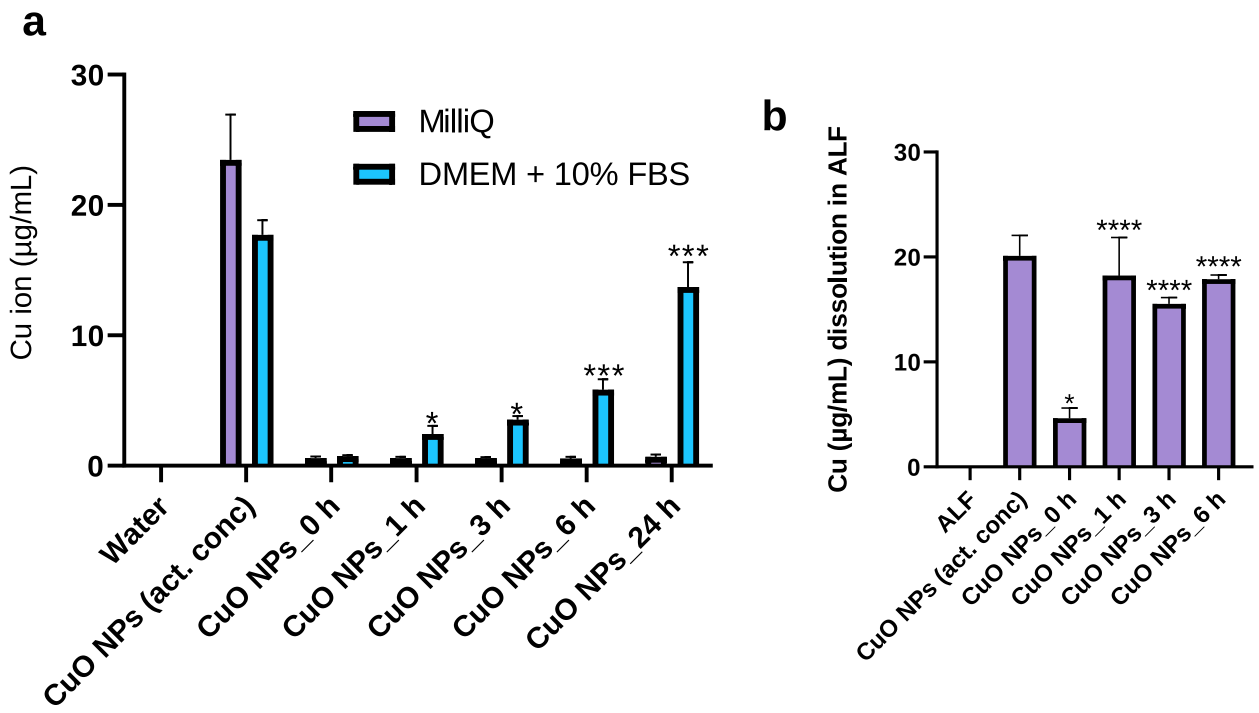

Figure S1. Dissolution of CuO NPs in biological media. (a) Time-dependent dissolution of CuO NPs in cell culture medium. No dissolution observed in Milli-Q® water. (b) Dissolution of CuO NPs in artificial lysosomal fluid (ALF) (pH=4.5). The release of Cu was determined by ICP-MS as detailed in Methods. Data are shown as mean values ± S.D. of three independent experiments. $^{*}p<0.05$, $^{***}p<0.001$.

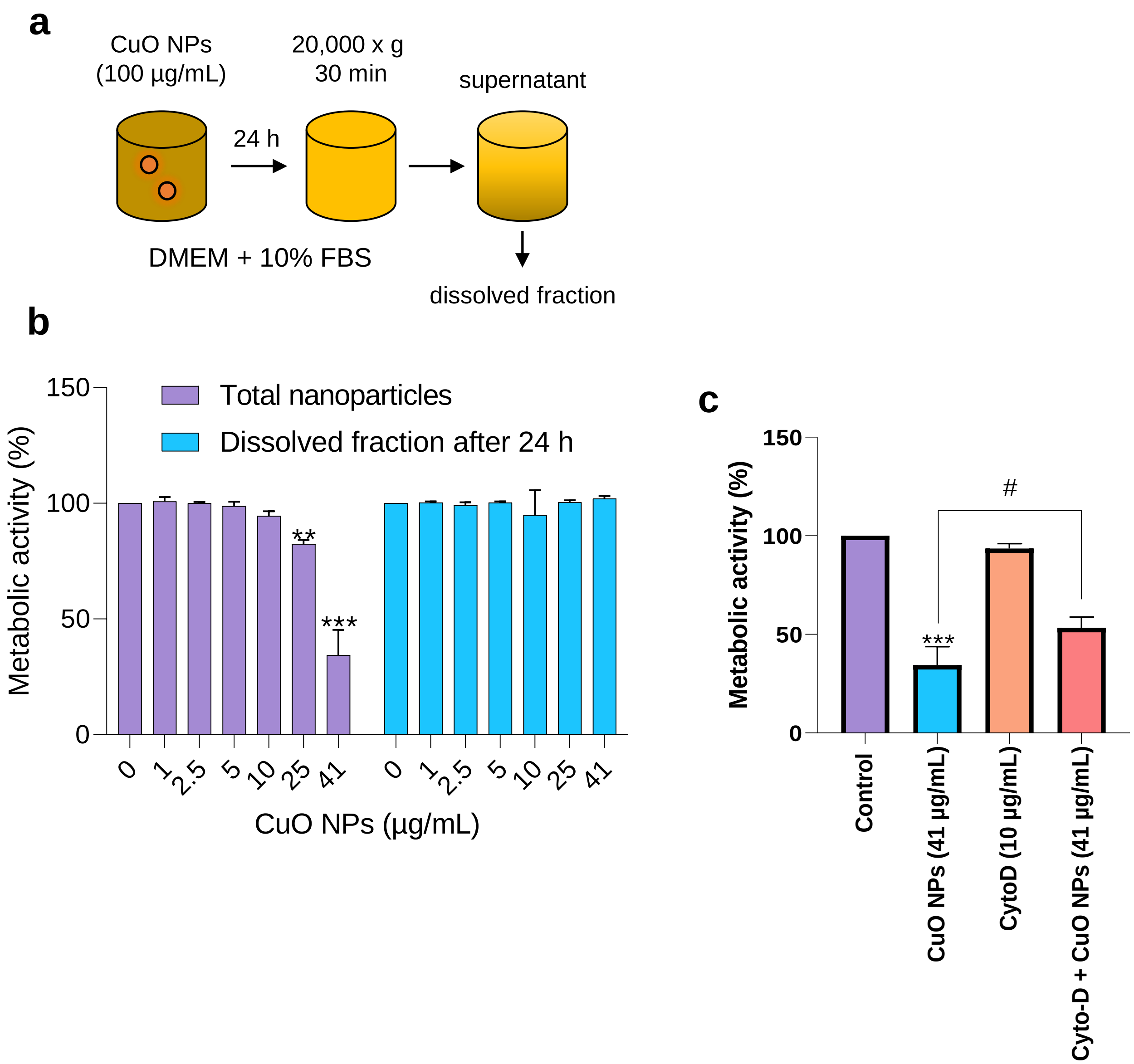

Figure S2. Cytotoxicity of intact CuO NPs *versus* the released Cu fraction. (a) The workflow for dissolution of CuO NPs in cell culture medium for 24 h prior to adding the released Cu fraction to cells. (b) Metabolic activity determined using the Alamar blue assay after 24 h of exposure to the CuO NPs added directy *versus* the dissolved fraction. (c) Metabolic activity of RAW264.7 cells exposed to CuO NPs in the presence or absence of cytochalasin D. Data shown are mean values ± S.D. of three independent experiments. **$p<0.01$, ***$p<0.001$, $^{\#}p<0.05$.

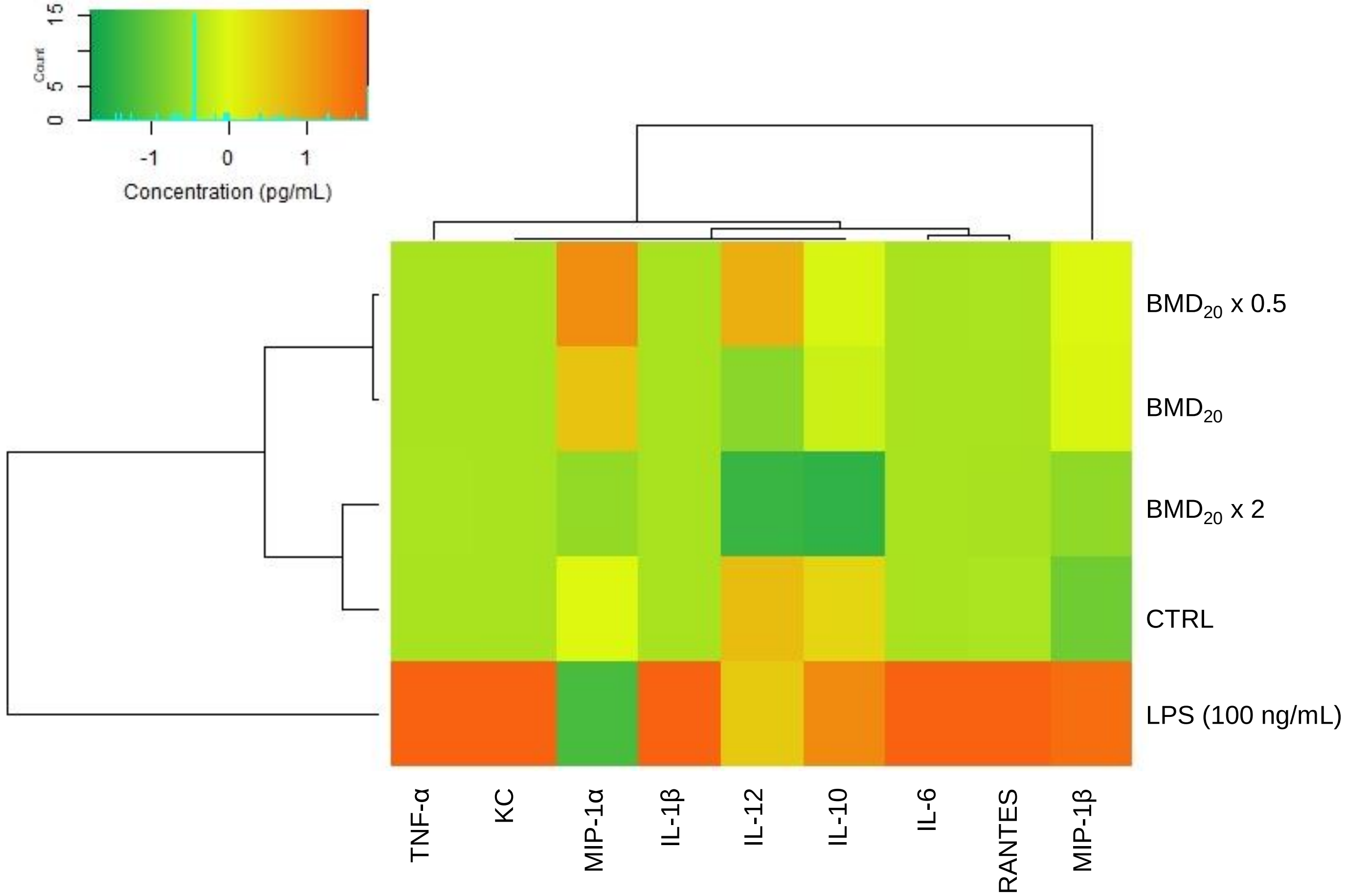

Figure S3. Cytokine release after exposure of cells for 24 h to CuO NPs. Cells were exposed to NPs at concentrations corresponding to BMD_{20}x0.5, BMD20, and BMD_{20}x2. LPS was included as a positive control. The secretion of cytokines and chemokines was quantified using the Bio-Plex® multiplex system (BioRad). Heat map generated by hierarchical cluster analysis. MCP-1 was excluded (below detection limit).

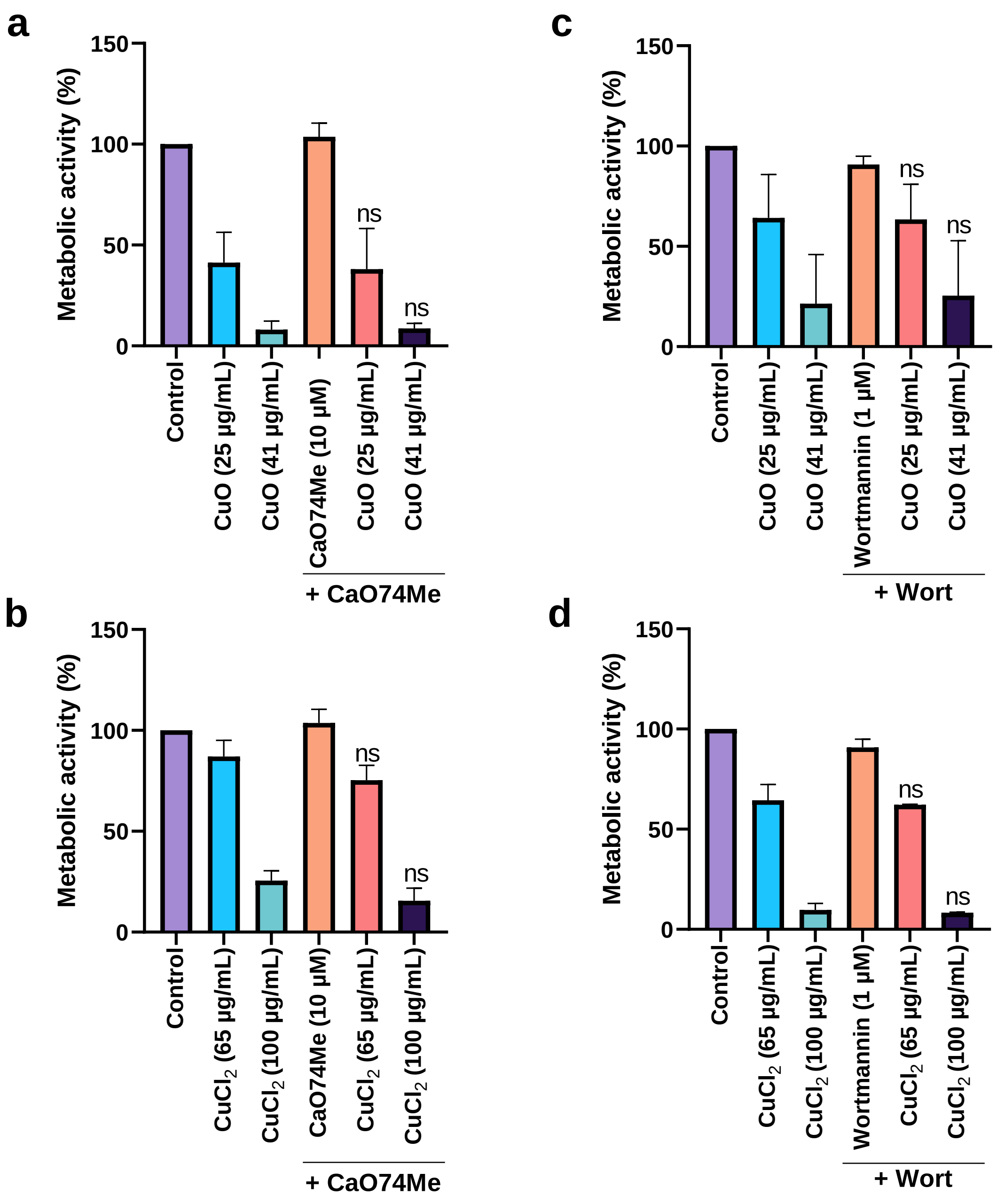

Figure S4. Effect of inhibitors on cell death elicited by CuO NPs or $CuCl_2$. (a-b) RAW264.7 cells were preincubated with the cathepsin B inhibitor, Ca-O74 Me (10 µM), and exposed to CuO NPs (a) or $CuCl_2$ (b). (c-d) Cells were preincubated with the PI3K inhibitor, wortmannin (1 µM), and exposed to CuO NPs (c) or $CuCl_2$ (d). Cell viability was determined using the Alamar blue assay. Data presented are mean values ± S.D. of three independent experiments. ns, not significant with respect to the corresponding treatment without inhibitor.

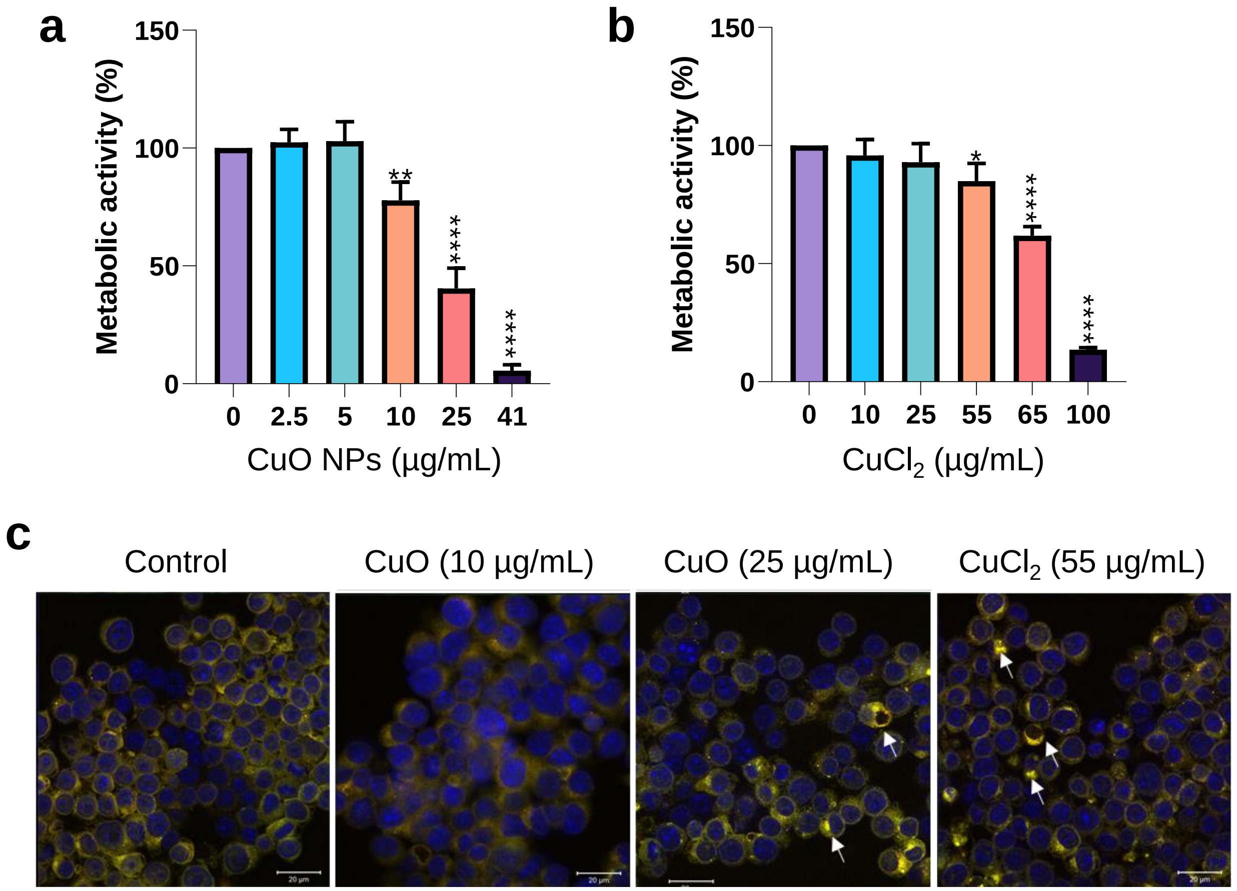

Figure S5. Autophagy induction. (a-b) Cell viability (metabolic capacity) of RAW-Difluo™ mLC3 cells after exposure for 12 h to CuO NPs (a), and $CuCl_2$ (b). Data are mean values ± S.D. of three independent experiments. *p<0.05, **p<0.01, ****p<0.0001 (significant difference between control and treatments). (c) Confocal micrographs of RAW-Difluo™ mLC3 cells exposed for 12 h to CuO NPs or $CuCl_2$.

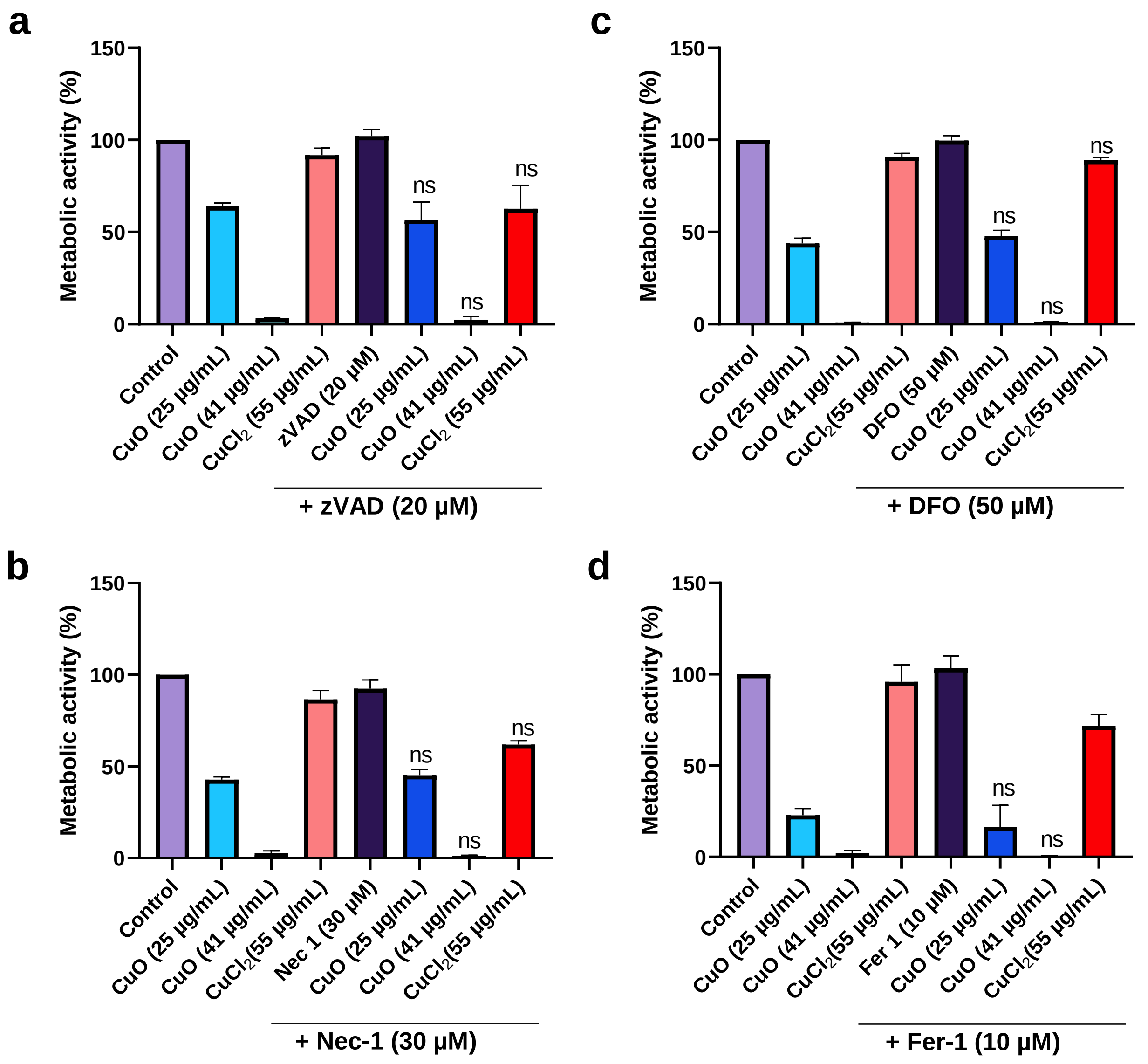

Figure S6. Pharmacological inhibitors of apoptosis, necroptosis, and ferroptosis failed to protect cells from CuO NPs or $CuCl_2$. The metabolic activity (indicative of cell viability) was determined using the Alamar blue assay at 24 h. Cells were preincubated with (a) zVAD-fmk, (b) necrostatin-1, (c) DFO, and (d) ferrostatin-1 prior to exposure to CuO NPs or $CuCl_2$ at the indicated concentrations. Data shown are mean values ± S.D. of three independent experiments. ns, not significant with respect to the corresponding treatment without inhibitor.

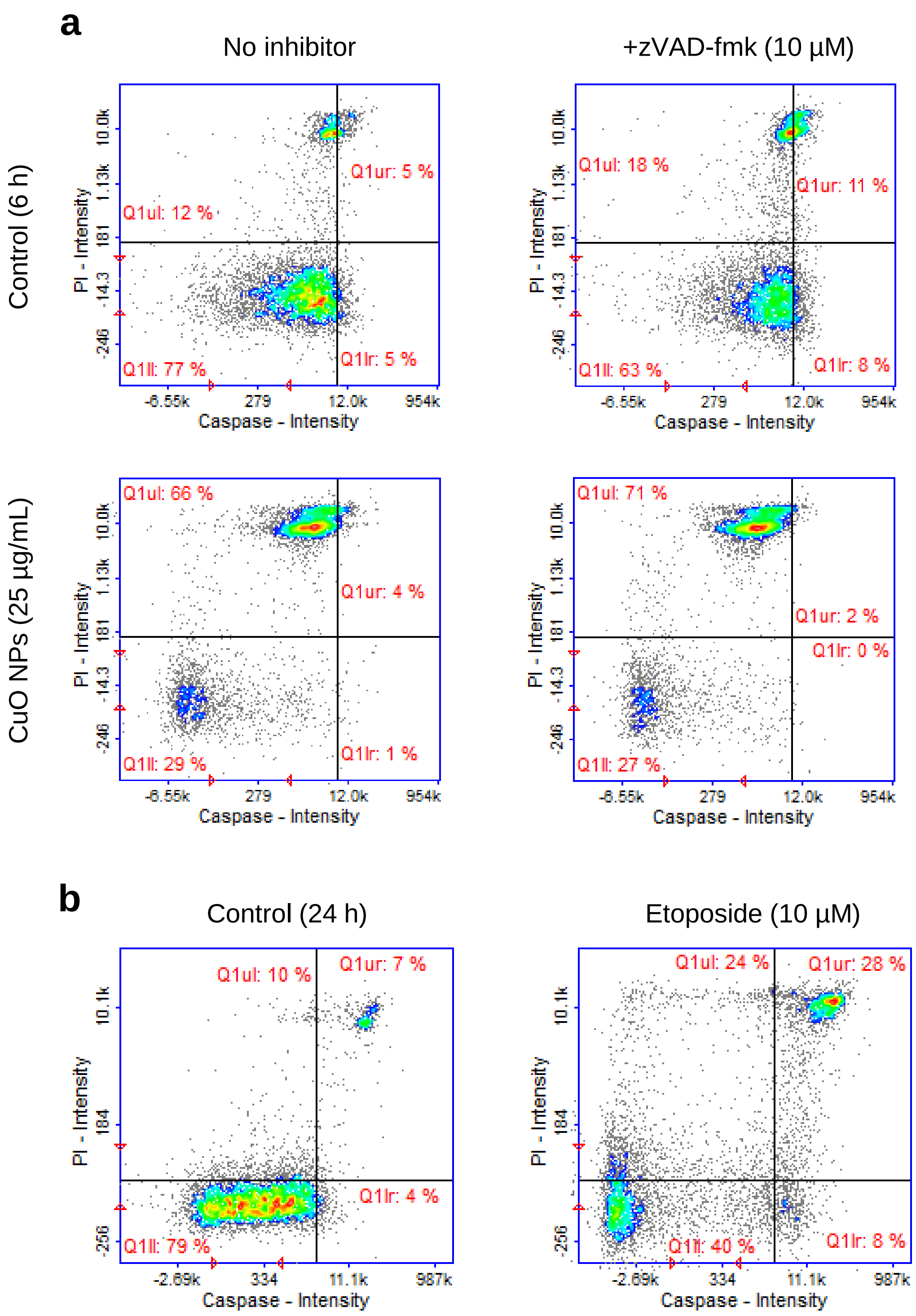

Figure S7. Caspase activation was determined using the FLICA™ affinity labeling method following exposure of RAW264.7 macrophages to (a) CuO NPs at the BMD_{20} dose (25 µg/mL) in the presence or absence of the pan-caspase inhibitor, zVAD-fmk. Cells were exposed for 6 h. Cells were co-stained using propidium iodide (PI) to assess for membrane integrity (necrosis). Cells were exposed to (b) etoposide for 24 h as a positive control.

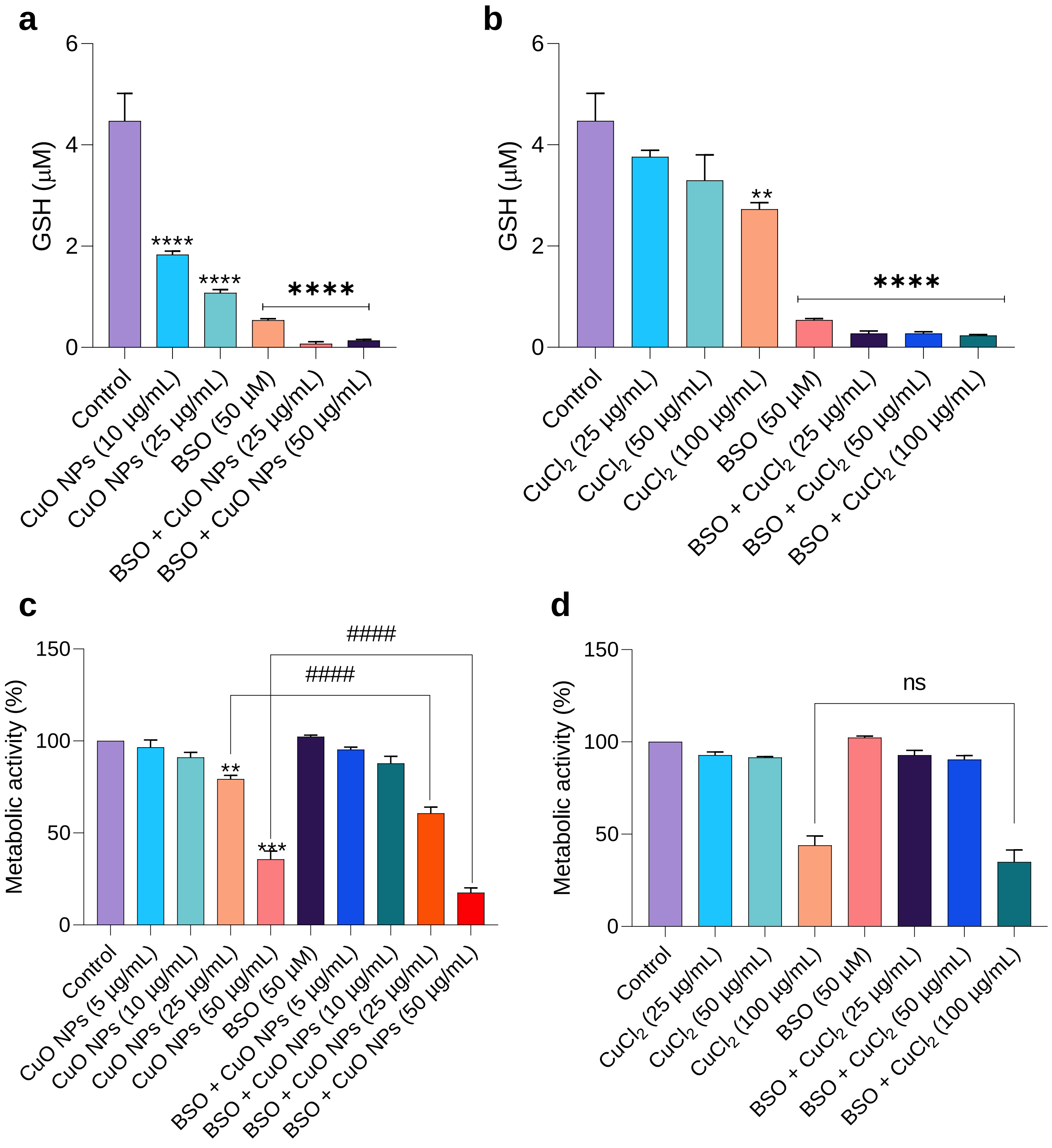

Figure S8. Cellular glutathione (GSH) levels following pre-treatment with the GSH-depleting agent BSO. (a,b) GSH content in RAW264.7 cells after exposure for 12 h to CuO NPs (a), and $CuCl_2$ (b) with and without BSO at the indicated concentrations. Cells were preincubated with BSO for 2 h. Data shown are mean values ± S.D. (n=3). $^{**}p<0.01$, $^{****}p<0.0001$ (significant difference between control and treatments). (d-e) Depletion of cellular GSH using BSO potentiated the toxicity of CuO NPs (d), while no differences in toxicity (as determined by using the Alamar Blue assay) was observed for $CuCl_2$ (e). Data shown are mean values ± S.D. (n=3). $^{**}p<0.01$, $^{***}p<0.001$ (significant difference between control and treatments); $^{\#\#}p<0.01$, $^{\#\#\#\#}p<0.0001$ (significant difference between the respective treatments).

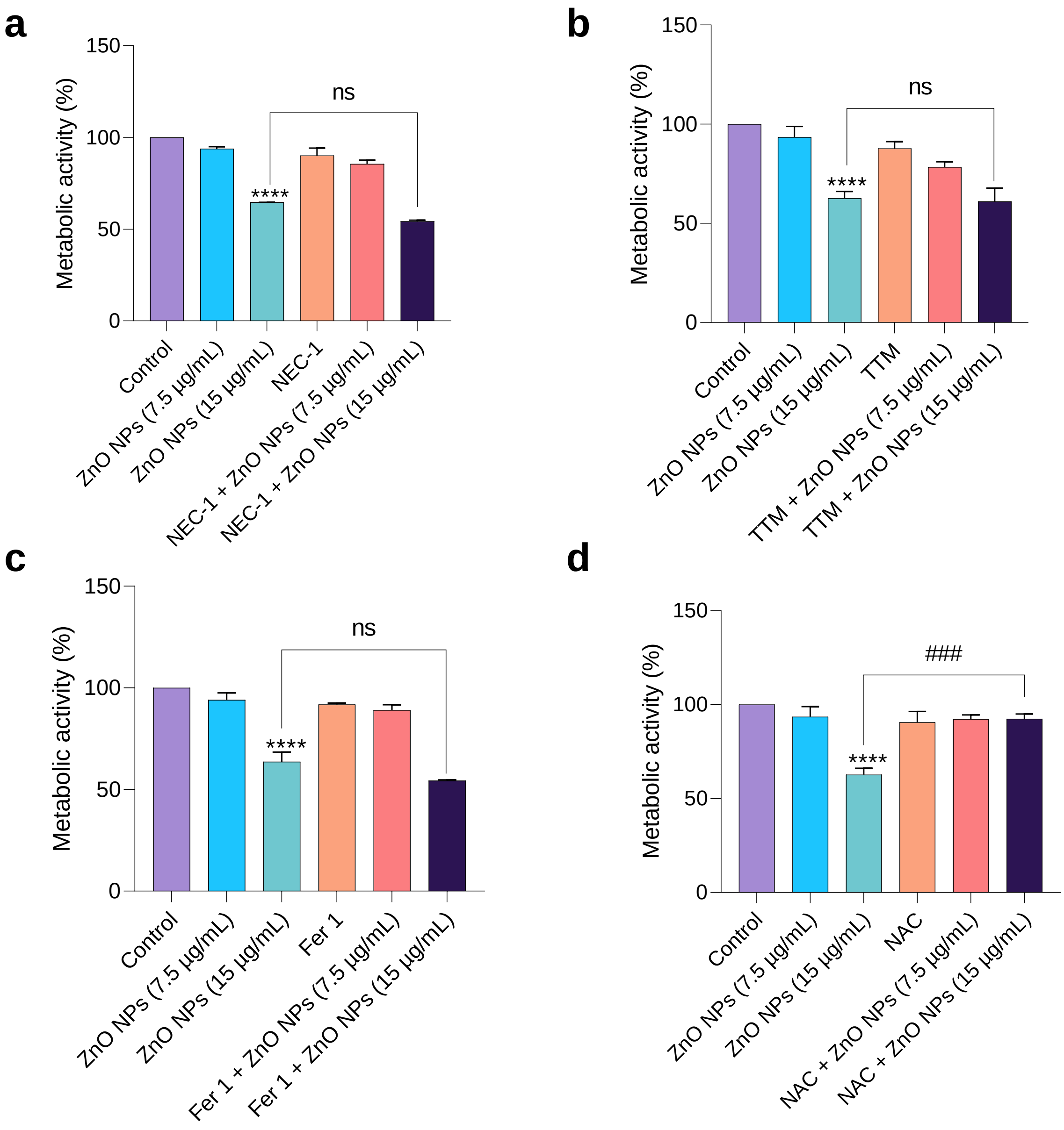

Figure S9. Pharmacological inhibitors of necroptosis and ferroptosis failed to protect against ZnO NP-triggered cell death. Cell viability (metabolic activity) was determined using the Alamar blue assay after 24 h of exposure. Cells were preincubated with (a) necrostatin-1 (30 µM), (b) the copper-chelating agent, TTM (50 µM), and (c) ferrostatin-1 (10 µM) prior to exposure to ZnO NPs. (d) Pre-incubation with N-acetyl cysteine (NAC) (1.25 mM) protected from ZnO NPs-triggered cell death. Data shown are mean values ± S.D. (n=3). ****p<0.0001, (significant difference between control and treatments); ###p<0.001 (significant difference between the treatments); ns, not significant.

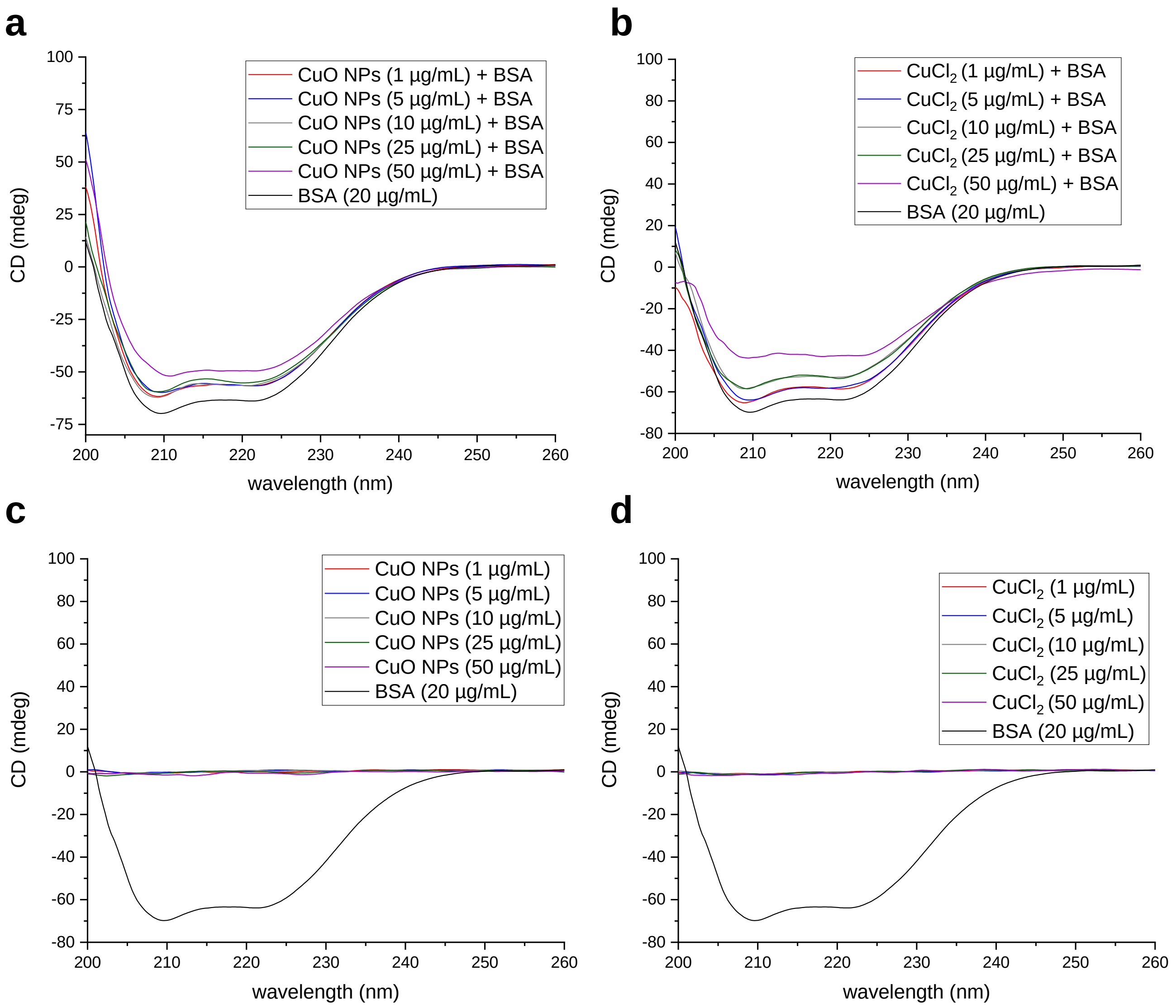

Figure S10. CuO NPs and $CuCl_2$ trigger misfolding of bovine serum albumin (BSA). CD spectroscopy of BSA (20 µg/mL) after incubation for 1 h with CuO NPs (a) and $CuCl_2$ (b) at the indicated concentrations. (c,d) CuO NPs and $CuCl_2$ alone did not interfere with the measurements.
